# Evidence for Integrin – Venus Kinase Receptor 1 Alliance in the Ovary of *Schistosoma mansoni* Females Controlling Cell Survival

**DOI:** 10.1371/journal.ppat.1006147

**Published:** 2017-01-23

**Authors:** Verena Gelmedin, Marion Morel, Steffen Hahnel, Katia Cailliau, Colette Dissous, Christoph G. Grevelding

**Affiliations:** 1 Institute for Parasitology, Justus-Liebig-University, Giessen, Germany; 2 CIIL – Center for Infection and Immunity of Lille Inserm U1019 - CNRS UMR 8204, University Lille, Lille, France; 3 UGSF - Unité de Glycobiologie Structurale et Fonctionnelle, CNRS UMR 8576, University Lille, Lille, France; George Washington University School of Medicine and Health Sciences, UNITED STATES

## Abstract

In metazoan integrin signaling is an important process of mediating extracellular and intracellular communication processes. This can be achieved by cooperation of integrins with growth factor receptors (GFRs). *Schistosoma mansoni* is a helminth parasite inducing schistosomiasis, an infectious disease of worldwide significance for humans and animals. First studies on schistosome integrins revealed their role in reproductive processes, being involved in spermatogenesis and oogenesis. With respect to the roles of eggs for maintaining the parasite´s life cycle and for inducing the pathology of schistosomiasis, elucidating reproductive processes is of high importance. Here we studied the interaction of the integrin receptor Smβ-Int1 with the venus kinase receptor SmVKR1 in *S*. *mansoni*. To this end we cloned and characterized SmILK, SmPINCH, and SmNck2, three putative bridging molecules for their role in mediating Smβ-Int1/SmVKR1 cooperation. Phylogenetic analyses showed that these molecules form clusters that are specific for parasitic platyhelminths as it was shown for integrins before. Transcripts of all genes colocalized in the ovary. In *Xenopus* oocytes germinal vesicle breakdown (GVBD) was only induced if all members were simultaneously expressed. Coimmunoprecipitation results suggest that a Smβ-Int1-SmILK-SmPINCH-SmNck2-SmVKR1 complex can be formed leading to the phosphorylation and activation of SmVKR1. These results indicate that SmVKR1 can be activated in a ligand-independent manner by receptor-complex interaction. RNAi and inhibitor studies to knock-down SmILK as a representative complex member concurrently revealed effects on the extracellular matrix surrounding the ovary and oocyte localization within the ovary, oocyte survival, and egg production. By TUNEL assays, confocal laser scanning microscopy (CLSM), Caspase-3 assay, and transcript profiling of the pro-apoptotic BCL-2 family members BAK/BAX we obtained first evidence for roles of this signaling complex in mediating cell death in immature and primary oocytes. These results suggest that the Smβ-Int1/SmVKR1 signaling complex is important for differentiation and survival in oocytes of paired schistosomes.

## Author Summary

Parasites of the genus *Schistosoma* cause schistosomiasis, a life-threatening infectious disease for humans and animals worldwide. Among the remarkable biological features of schistosomes is the differentiation of the female gonads which is controlled by pairing with the male and a prerequisite for egg production. Eggs, however, are not only important for the maintenance of the life-cycle; they also cause the pathological consequences of schistosomiasis. Part of the eggs gets trapped in host tissues such as liver and spleen and trigger inflammatory processes, finally leading to liver cirrhosis. Research activities of the last decade have indicated that different families of cellular and receptor-type kinases but also integrins contribute to the control of mitogenic activity and differentiation the female goands. In this context an unusual class of receptor tyrosine kinases (RTKs) has been identified, the venus kinase receptors (SmVKRs). By biochemical and molecular approaches we demonstrate that SmVKR1 activation can be achieved by cooperation with a signaling complex consisting of the beta integrin receptor Smβ-Int1 and the bridging molecules SmILK, SmPINCH, SmNck2. Besides unravelling a novel way of SmVKR1 activation, we provide evidence that this complex controls the differentiation status of oocytes by regulating cell death-associated processes.

## Introduction

Communication of cells with their environment is an essential requirement to regulate fundamental biological processes such as cell growth and differentiation. Different types of membrane-linked receptors mediate these communication processes, sometimes in a solitary, single receptor-mediated way, sometimes in a cooperative, multiple receptors-mediated way. The latter leads to the integration of different signaling cascades to execute one or more complex operations [1–4].

Schistosomes are parasitic platyhelminths causing schistosomiasis, one of the most threating infectious diseases worldwide after malaria [5–7]. As the only members of the trematodes, schistosomes have evolved separate sexes. The pathology of the disease is caused by eggs which are produced by paired schistosome females in the final host. Egg production is a complex process that involves not only the participation of different cell types, oocytes and vitellocytes. It also comprises the participation of different organs, ovary and vitellarium, whose development in the female depends on a close and permanent pairing contact with the male [8–11]. Although this nearly unique way of regulating sexual development in the animal kingdom is long known [12] and fundamental for the reproductive biology of schistosomes as well as for the pathogenic consequences of schistosomiasis, understanding the underlying molecular principles is still in its infancy.

A number of signaling cascades have been uncovered that are involved in the control of gonad differentiation in paired schistosome females [13, 14]. In *S*. *mansoni*, a kinase complex of three different cellular tyrosine kinases (CTKs) was postulated, whose members were able to interact with different receptors such as β integrin (Smβ-Int1) and venus kinase receptors (SmVKRs) [15–18). The latter represent an unusual type of receptor tyrosine kinases (RTKs) consisting of an intracellular tyrosine kinase (TK) domain with homology to that of insulin receptors (IR) and an extracellular venus-flytrap (VFT) module, whose structure is similar to the ligand binding domain of G protein-coupled receptors (GPCRs) of the C class [19, 20]. RNAi-mediated knockdown of Smβ-Int1 and SmVKRs exhibited their roles in oogenesis and egg formation of *S*. *mansoni* females [17, 21]. As potential ligands, L-Arginine (L-Arg) and calcium ions were discovered, which activated SmVKR1 and SmVKR2, respectively, when they were expressed in *Xenopus* oocytes [22]. Studies in *Aedes aegypti* have substantiated roles of VKRs for reproduction. AaeVKR expression was found in the ovaries of blood-fed adult females and its activation by the neuroparsin, ovary ecdysteroidogenic hormone, was demonstrated [23]. Since neuroparsins are neuropeptides specific for arthropods [24] it still remains elusive whether and which other molecules except ions and amino acids may be able to activate schistosome VKRs [25].

Physical associations were documented between integrins and GFRs [26]. The latter include RTKs, whose activities can be likewise influenced by integrins [27, 28]. As shown in skin fibroblasts, interactions with integrins support the activation of the GFRs even in the absence of a ligand [29]. Among other functions the αvβ3 integrin was found to directly associate with the insulin-like IGF1 receptor in vascular cells [30]. Such integrin-GFR interactions are mediated by bridging molecules such as ILK (*i*ntegrin-*l*inked *k*inase), PINCH (*p*articularly *i*nteresting *n*ew *c*ysteine-*h*istidine-rich protein) and Nck2 (*n*on-*c*atalytic region of tyrosine *k*inase adaptor protein). They are central parts of an integrin-actin hub mediating many protein interactions that regulate processes such as pericellular matrix deposition, cell morphology, motility and apoptosis [31–33].

Aims of our study were to investigate whether Smβ-Int1 and SmVKR1, which colocalize in the ovary of *S*. *mansoni* females and whose RNAi-mediated knock-downs led to similar phenotypes [17, 21], may interact to govern differentiation processes in this organ. Our findings provide first evidence for this cooperation and for a Smβ-Int1-induced activation of SmVKR1, which is independent from an extracellular VKR ligand. Furthermore, our data suggest that Smβ-Int1/SmVKR1 cooperatively control the differentiation status of oocytes by regulating cell death-associated processes.

## Results

### Cloning and sequence analyses of SmILK, SmPINCH and SmNck2

In eukaryotic systems integrin-GFR cooperation can be accomplished by ILK, PINCH, and Nck2. As cytoplasmic molecules they bind to the intracellular parts of integrin (ILK) or GFR (Nck2), or simultaneously to both receptors with PINCH as bridging molecule connecting ILK and Nck2 [32, 33]. To investigate the possibility of such an interaction in *S*. *mansoni*, we first searched for orthologs in the schistosome database [34, 35]. Based on comparisons to orthologs from human, potential candidate genes were identified and analyzed *in silico*. Deletion clones, including those potentially originating from alternative splicing events, were excluded from further analyses. Finally, full-length cDNAs of the longest variants of SmILK (Smp_079760), SmPINCH (Smp_020540.2), and SmNck2 (Smp_014850) were amplified by RT-PCR, cloned, and sequenced.

Detailed sequence analyses showed the cloned cDNAs of SmILK, SmPINCH, and SmNck2 isolated from the Liberian strain of *S*. *mansoni* [36] were 100% identical to those of the Puerto Rican strain used for genome sequencing [34, 35, 37]. BLAST analyses showed 97% and 89% identity at the cDNA level to ILK orthologs of *S*. *haematobium* (XM_012945977.1) and *S*. *japonicum* (AY810458.1), respectively. Furthermore, SmILK exhibited all typical domains for this class of enzymes such as three N-terminal ankyrin repeat domains as well as one C-terminal kinase-like domain (S1A Fig). The latter is considered as a catalytically inactive domain, which makes ILK a potential pseudokinase without catalytic but with structural importance [38, 39].

As zinc-finger adaptor protein, PINCH contains five Lim (similar to *L*in11, *I*sl-1 and *M*ec-3 proteins) domains including eight zinc-binding residues [40]. SmPINCH follows this characteristic structural organization (S2A Fig). At the cDNA level SmPINCH showed 91% and 81% identity to PINCH orthologs of *S*. *haematobium* (XP_012797616.1) and *S*. *japonicum* (AAX26687.2), respectively.

Nck2, finally, represents another adaptor protein consisting of three SH3-domains and one C-terminal SH2-domain. The latter is important for binding to GFRs whereas one or more of the SH3-domains can support GFR binding or mediate interactions to downstream partners such as PINCH [41]. The occurrence of all these domains at comparable positions (S3A Fig) indicated that SmNck2 is an ortholog of Nck2 proteins. BLAST analyses showed 94% and 85% identity of SmNck2 at the cDNA level to Nck2 orthologs of *S*. *haematobium* (XM_012936558.1) / *S*. *japonicum* (AY809191.1), respectively.

Phylogenetic analyses of the three molecules with orthologs of vertebrates and invertebrates demonstrated that the schistosome ILK, and Nck2 formed separate clusters together with other parasitic platyhelminths, and schistosome Nck2 was part of a trematode cluster separate from the cestodes and other invertebrates (S1B–S3B Figs). This observation coincides with previous findings made for the schistosome α and β integrins, which according to phylogenetic analyses constitute parasite-specific clades separate from free-living flatworms and further metazoan integrins [17].

### SmILK, SmPINCH and SmNck2 are transcribed in the reproductive organs

**Fig 1:**
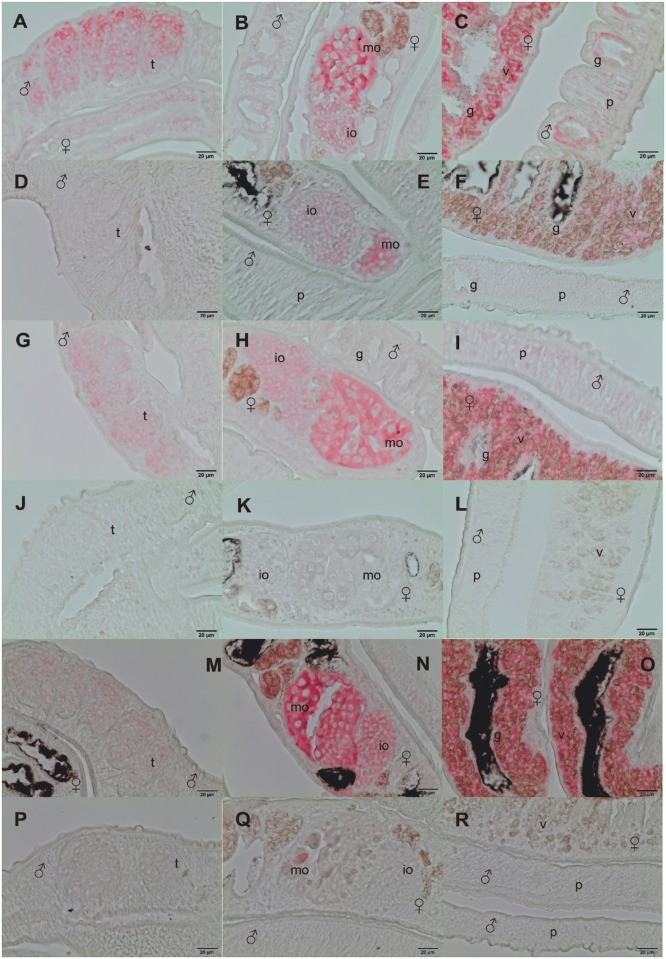
*In situ*-hybridization localize transcripts of SmILK, SmPINCH and SmNck2 in the gonads of *S*. *mansoni*. Representative sections (5 μm) of schistosome couples are shown (males and females are indicated by biological symbols), which were hybridized with DIG-labeled antisense-RNA probes of SmILK (A–C), SmPINCH (G–I), SmNck2 (M-O). mRNA transcripts of SmILK were detected in the testes (t; see A), in mature (mo) and immature (io;) oocytes within the ovary (see B), the gastrodermis (g; see C), the parenchyma (p; see A-C) and the vitellarium (v; see C). Similar patterns were found for SmPINCH (testes, G; ovary, H; vitellarium and parenchyma, I) and SmNck2 (testes, M; ovary N; vitellarium, O). For control, sense-RNA probes were used for hybridization (SmILK, D-F: showing testes, ovary, vitellarium, gastrodermis and parenchyma, respectively; SmPINCH, J-L: showing testes, ovary, vitellarium, gastrodermis and parenchyma, respectively; SmNck2, P-R: showing testes, ovary, vitellarium, gastrodermis and parenchyma, respectively). Scale bars are 20 μm as indicated.

*In situ*-hybridization localized the transcripts of SmILK, SmPINCH and SmNck2 in the ovary and the vitellarium of the female as well as in the testis of the male (Fig 1). Ovary and testis transcription were independently confirmed by gonad RNA-specific RT-PCRs [42] showing amplification products of the expected sizes (S4 Fig). In each case the *in situ*-hybridization signals appeared to be stronger in the large part of the bulb-like ovary which contains mature primary oocytes. Furthermore, SmILK and SmPINCH transcripts were found in the parenchyma of both genders and in the subtegumental area, within the gastrodermis of males, and around the ootype, although not as dominant as in the gonads. Sense transcripts of all three genes as controls showed varying degrees of week signals (very low in case of SmNck2). This indicates antisense regulation, a finding made for different schistosome genes before including integrins and further molecules involved in reproduction [17, 43, 44].

### Functional studies in *Xenopus* oocytes reveal Smβ-Int1—SmVKR1 complex formation-dependent activation of the GVBD

To elucidate the roles of SmILK, SmPINCH, and SmNck2 in complex formation with Smβ-Int1 and SmVKR1, we started a series of biochemical experiments in *Xenopus* oocytes. Previous studies had demonstrated the efficiency of expression of schistosome genes in this system and, furthermore, the possibility to study kinase activities by their capacities to induce resumption of meiosis and germinal vesicle breakdown (GVBD) [15, 45]. Activation by L-Arg of the SmVKR1 kinase led to GVBD, which failed when a dead-kinase mutant of SmVKR1 was used [22].

No GVBD was observed in *Xenopus* oocytes when a wildtype form of SmILK was expressed (Table 1). This is in agreement with the present view that SmILK may represent a pseudokinase ortholog of eukaryote ILKs lacking catalytic activity [38, 39]. Also PINCH and Smβ-Int1 failed to induce oocyte maturation. According to previously published data [22], the wildtype form of SmVKR1 induced 90% GVBD only in the presence of its activating ligand L-Arg. The constitutively active SmVKR1 mutant induced GVBD, whereas a dead kinase mutant did not.

**Table 1:** 

Inj	SmβInt	SmILK		SmPINCH		SmNck2		SmVKR1			GVBD	GVBD + L-Arg
		wt	del	wt	del	wt	del	wt	ca	dk	%	%
**1**		+									0	
**2**				+							0	
**3**	+										0	
**4**								+			0	90
**5**									+		90	
**6**										+		0
**7**	+	+		+							0	
**8**		+		+				+			0	90
**9**	+			+				+			0	100
**10**	+	+						+			0	90
**11**	+	+		+				+			**80**	90
**12**	+		+	+				+			0	50
**13**	+	+*QLT		+		+		+			0	
**14**	+	+			+			+			0	90
**15**	+	+		+			+	+			10	
**16**	+	+		+					+		100	
**17**	+	+		+						+	0	
**18**	+	+		+				SmVKR2			0	
**19**	+	+		+				SER			0	
**20**	+	+		+				SmIR1			0	
**21**	+	+		+				SmIR2			0	

Summarized are 21 transfection experiments in *Xenopus* oocytes and germinal vesicle breakdown (GVBD, in %) as physiological assay with different constructs alone or in combination (indicated by single or multiple “+”-signs). Inj, injection experiment number; SmβInt = Smβ-Int1; wt, wildtype; del, deletion mutant (SmILK: SmILKΔAnk1; SmPINCH: SmPINCHΔLIM4; SmNck2: SmNck2ΔSH3; SmVKR1 dk, dead kinase [22]). Injections 18–21, control experiments in which SmVKR1 was replaced by SmVKR2 [22], SER (*S*. *mansoni* EGF Receptor; [45, 46]), or the insulin receptor orthologs SmIR 1 and SmIR2 [47]. In these cases no GVBD was induced (compare to Inj 11). ca, constitutively active (SmVKR1, SmVKR1^YYRE^ [22]); GVBD values (%) were determined as previously described and represent the mean of two independent experiments with 10 oocytes each [15, 45];

*QLT, inhibitor of the integrin-linked kinase (1 μM); L-Arg, L-Arginine; empty boxes in the last two columns: not determined.

When Smβ-Int1, SmILK, and SmPINCH were coexpressed, no GVBD was observed. However, when these three proteins were co-expressed with SmVKR1, GVBD was obtained independently of the addition of L-Arg (Table 1, Inj 11). This suggested that SmVKR1 kinase activation could be induced by its participation to the complex with Smβ-Int1, SmILK and SmPINCH. However, in this injection (no. 11) GVBD was activated in the absence of SmNck2. This finding led to the questions whether *S*. *mansoni* Nck2 is dispensable for complex formation, or whether *Xenopus* Nck2 may have rescued complex formation in this case? When deletion mutants of SmILK (SmILKΔAnk1, missing the first ankyrin repeat necessary for interaction with PINCH; [32, 33]) or SmPINCH (SmPINCHΔLIM4, missing the fourth Lim domain necessary for interaction with Nck2/GFR; [32, 33]) or SmNck2 (SmNck2ΔSH3, missing the SH3 domain necessary for interaction with PINCH; [33]) were used, activation of SmVKR1 was no more observed. The result with the deletion mutant of SmNck2 (Table 1, Inj 15) indirectly indicated the presence of *Xenopus* Nck2 in the complex and a competitive situation between *Xenopus* Nck2 and SmNck2ΔSH3 when the latter protein was present. Furthermore, adding the ILK-inhibitor QLT-0267 (1 μM) also prevented GVBD in oocytes expressing the wildtype forms of Smβ-Int1, SmILK, SmPINCH, SmNck2, and SmVKR1. These data suggest a direct interaction of these proteins, and also that Smβ-Int1-SmILK-SmPINCH-SmNck2-SmVKR1 complex formation is able to induce GVBD in *Xenopus* oocytes in the absence of a ligand for SmVKR1. This interaction appeared to be specific for SmVKR1, since other RTKs such as SmVKR2 [22], SER (*S*. *mansoni* EGF Receptor; [45, 46] or the insulin receptor orthologs SmIR 1 and SmIR2 [47] were not activated by this complex. Furthermore, since GVBD was supposed to be dependent on the kinase activation of SmVKR1, we checked the autophosphorylation status of SmVKR1 by Western blot analysis. GVBD occurred only when SmVKR1 was phosphorylated (see below).

### Smβ-Int1/SmVKR1 complex formation leads to ligand-independent SmVKR1 phosphorylation

**Fig 2:**
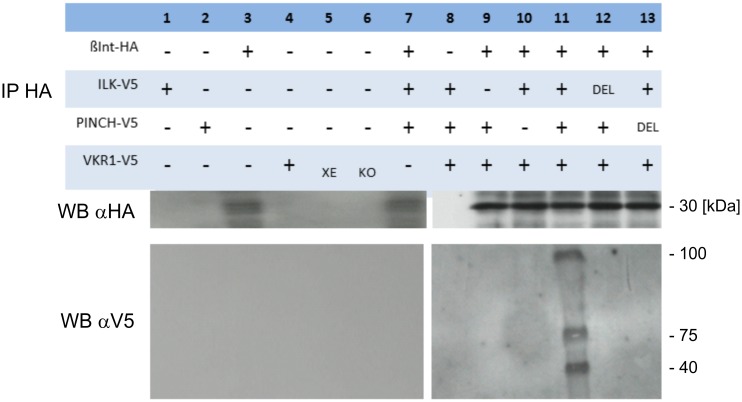
Co-Immunoprecipitation confirms complex formation. Co-Immunoprecipitation of HA-tagged Smβ-Int1 and V5-tagged SmILK (about 56 kDa), SmPINCH (about 42 kDa), and SmVKR1 (about 150 kDa) expressed in *Xenopus* oocytes. Anti-HA antibodies immunoprecipitated Smβ-Int1 together with SmILK, SmPINCH, and SmVKR1 upon co-expression in oocytes only when their wildtype forms were used (lane 11). Using deletion variants (DEL) of SmILK or SmPINCH, no immunoprecipitated interaction partners were detected (lanes 12, 13). No immunoprecipitated molecules were detected in further controls including individual molecules (lanes 1–6) or combinations with three of the four potential binding partners (lanes 7–10).

**Fig 3:**
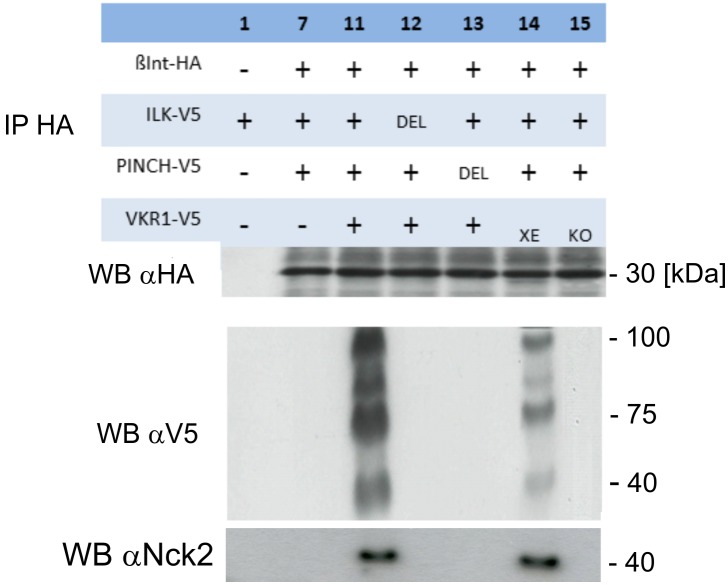
Complex formation occurs with the constitutively active SmVKR1 variant but not with a dead-kinase mutant. Co-Immunoprecipitation of HA-tagged Smβ-Int1 (β-Int-HA) and a selection (see Fig 2; the same numbering was used, except lanes 14, 15) of combinations of V5-tagged SmILK (ILK-V5), SmPINCH (PINCH-V5), and SmVKR1 (VKR1-V5) constructs in *Xenopus* oocytes. Anti-HA antibodies immunoprecipitated Smβ-Int1 together with SmILK, SmPINCH, endogenous Nck2, and SmVKR1 upon co-expression in oocytes only when their wildtype forms (lane 11) or the constitutively active form of SmVKR1^XE^ (lane 14) were used. In these cases *Xenopus* Nck2 was part of the complex formation as detected in lanes 11 and 14 by anti-human Nck2 antibody. Using deletion variants (DEL) of SmILK (lane 12) and SmPINCH (lane 13), SmILK individually (lane 1), a combination of wildtype SmILK, SmPINCH and Smβ-Int1 (lane 7), or a dead kinase variant (KO) of SmVKR1 (lane 15), no immunoprecipitated interaction-partners were detected.

To confirm the existence and the function of this complex, the HA-tagged intracellular part of Smβ-Int1 [17] was co-expressed in *Xenopus* oocytes together with V5-tagged variants of SmILK (wildtype and SmILKΔAnk1), SmVKR1 (dead kinase and constitutively active mutants), or Flag-tagged SmPINCH (wildtype and SmPINCHΔLIM4) and SmNck2 (SmNck2ΔSH3) for co-immunoprecipitation. In this series of experiments, L-Arg was not used for stimulating SmVKR1 activity (Table 2). Besides investigating the GVBD-inducing activity of appropriate combinations of complex members in their wildtype or mutated forms, oocyte lysates were immunoprecipitated with an α-HA antibody, and Western blot analyses were performed with α-HA or α-V5 to investigate the presence of members complexed with Smβ-Int1. The results showed V5-tagged SmILK, SmPINCH, and SmVKR1 in HA-tagged precipitates only when the wildtype forms were used (Fig 2, lane 11). A replication of the experiment demonstrated complex formation also when the constitutively active SmVKR1 variant was used (Fig 3, lane 14). As expected, no V5-tagged precipitates were detected, when the deletion variants SmILKΔAnk1 or SmPINCHΔLIM4 or the dead kinase variant of SmVKR1 were used. These results corresponded to the GVBD results obtained, confirming the formation of a complex of these four proteins. However, complex formation was possible due to the presence of *Xenopus* Nck2 (Fig 3), which was detected by Western blot analysis. This confirmed the previous interpretation of the GVBD experiment (Table 1).

**Table 2:** Results of germinal vesicle breakdown assays in *Xenopus* oocytes and subsequent coimmunoprecipitation experiments.

Exp	SmβInt (HA)	SmILK (V5)	SmPINCH (V5)	SmNck2 (Flag)	SmVKR1 (V5)	GVBD (%)	SmVKR1 (phos)
**1**	+	+	+	+	+	90	yes
**2**	+	SmILKΔAnk1	+	+	+	0	no
**3**	+	+	SmPINCHΔLIM4	+	+	0	no
**4**	+	+	SmPINCHΔLIM1	+	+	0	no
**5**	+	+	+	SmNck2ΔSH3	+	10	no
**6**	+	+	+	+	SmVKR1dk	15	no
**7**	+	+ *QLT	+	+	+	0	no
**8**					SmVKR1ca	90	yes

Overview of the results 8 experiments (Exp) with different constructs alone or in combination (indicated by “+”), and without adding L-Arg. HA, V5, and Flag indicate the tags used for the appropriate molecules. It is indicated when wildtype (+) forms were replaced by mutant forms (see Table 1). GVBD (%), germinal vesicle breakdown frequency;

*QLT, inhibitor of the integrin-linked kinase (1 μM); phos = phosphorylation of SmVKR1; dk, dead kinase; ca, constitutively active [22].

**Fig 4:**
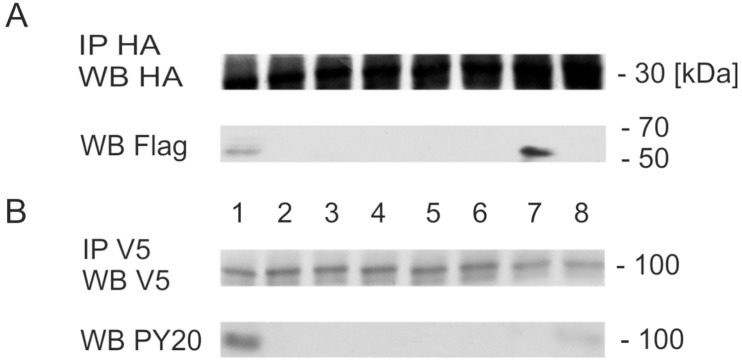
SmNck2 is part of the complex, and SmVKR1-phosphorylation occurs only when all complex-members are expressed. Western blot analysis of complex members expressed in *Xenopus* oocytes. (A) following transfection with SmVKR1, Smβ-Int1, SmILK, SmPINCH, and SmNck2 constructs (see below) coimmunoprecipitation with anti-HA and subsequent Western blot (WB) analyses with anti-HA (HA-tagged: Smβ-Int1) and anti-Flag (SmNck2ΔSH3) confirmed the presence of Nck2 in the complex. (B) following coimmunoprecipitation with anti-V5, Western blot (WB) analyses with anti-V5 (V5-tagged SmVKR1) and anti-PY20 confirmed tyrosine phosphorylation of SmVKR1 only in case of a combination with Smβ-Int1, SmILK, SmPINCH as well as SmNck2 and without ligand (L-Arg) addition (lane 1, WB PY20). When deletion mutants of SmILK (SmILKΔAnk1; lane 2), SmPINCH (SmPINCHΔLIM1, SmPINCHΔLIM4; lanes 3, 4), SmNck2 (SmNck2ΔSH3; lane 5), SmVKR1 (dk, dead kinase mutant; [22]; lane 6), or the ILK inhibitor QLT-0267 (1 μM; lane 7) were used, no tyrosine phosphorylation of SmVKR1 was detected. As a positive control, L-Arg was added as ligand, which induced SmVKR1 tyrosine phosphorylation as expected (lane 8).

To investigate SmVKR1 activation upon complex formation, the phosphorylation status of this receptor was investigated. After confirming that SmNck2 is also part of the immunoprecipitated protein complex (Fig 4A), Western blot analyses showed that SmVKR1 phosphorylation (without adding L-Arg) occurred only when it was coexpressed together with the wildtype forms of Smβ-Int1, SmILK, SmPINCH, and SmNck2 (Fig 4B). When deletion mutants of individual complex partners or the ILK inhibitor QLT-0267 were used, no SmVKR1 phosphorylation was detected. This is in perfect agreement with the GVBD results obtained with the same combinations of molecules (Table 1).

### Ligand-independent SmVKR1 activation leads to ERK, JNK, and Akt phosphorylation in *Xenopus* oocytes

**Fig 5:**
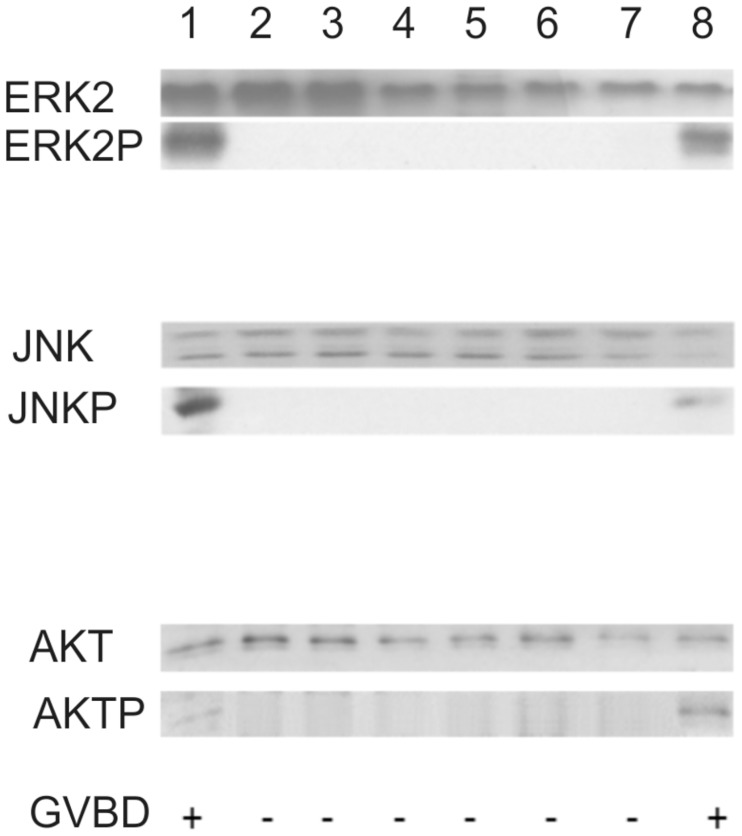
Complex formation induces ERK2, JNK and AKT pathways in *Xenopus* oocytes in a ligand-independent way. Western blot analyses of signaling pathways triggered by Smβ-Int1 complex-mediated SmVKR1 activation in *Xenopus* oocytes. The schistosome complex members were expressed in *Xenopus* oocytes for 5 h with or without ligand. Oocyte lysates were analyzed to investigate the phosphorylation state of ERK2, JNK and AKT following SmVKR1- Smβ-Int1 complex formation. In the absence of L-Arg as ligand, and in case of the combination of Smβ-Int1, SmILK, SmPINCH, SmNck2, and SmVKR1 (lane 1) phosphorylation of ERK2 (ERK2P), JNK (JNKP), and AKT (AKTP) was observed. In cases of using mutated forms (SmILKΔAnk1, lane 2; SmPINCHΔLIM1, lane 3; SmPINCHΔLIM4, lane 4; SmNck2ΔSH3, laneΔ 5; SmVKR1 KO, lane 6) or the ILK inhibitor QLT-0267 (1 μM; lane 7), no phosphorylation of ERK2, JNK, and AKT was detected. As expected, phosphorylation of ERK2, JNK, and AKT was observed in SmVKR1-expressing oocytes stimulated by L-Arg (1 μM), which served as positive control (lane 8).

In a previous study it was shown that upon SmVKR1 stimulation with L-Arg signaling pathways known to be involved in RTK signaling were activated in *Xenopus* oocytes. Among these were ERK, JNK, and Akt pathways [21]. To find out whether Smβ-Int1/SmVKR1 complex formation without L-Arg induction activates the same signaling cascades in *Xenopus* oocytes, we performed cotransfection experiments and subsequent phosphorylation assays. Indeed, the obtained results showed that SmVKR1 in cooperation with all complex partners induced the phosphorylation of ERK, JNK, and AKT in a ligand-independent manner (Fig 5). In analogy to the previous results, no phosphorylation of these signaling molecules was observed when one of the complex members was used in its mutated form or when the ILK-inhibitor QLT-0267 was applied. The effect of Smβ-Int1/SmVKR1 complex formation on the phosphorylation of ERK, JNK, and AKT resembled the activation of *Xenopus* oocyte receptors by insulin or the natural ligand progesterone.

### Affecting SmILK signaling by RNAi or inhibitor treatment influences egg production, ovary structure and oocyte integrity in schistosome females

**Fig 6:**
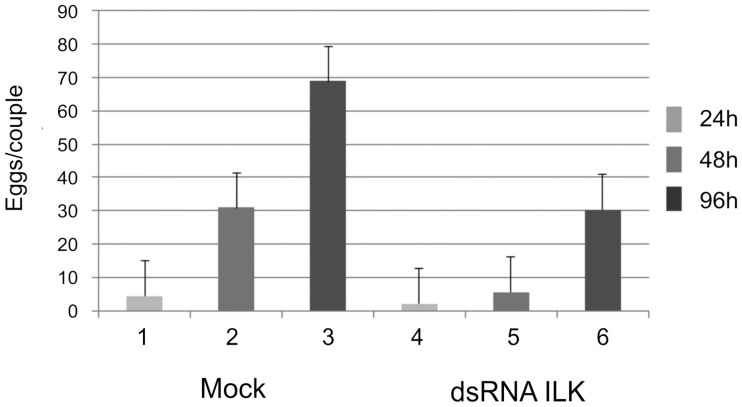
Egg production of worm couples decreases following RNAi suppressing SmILK activity *in vitro*. Compared to a MOCK control, egg production of couples treated with SmILK dsRNA significantly decreased from 48 h post treatment on.

Because SmILK is one of the decisive complex partners mediating Smβ-Int1 cooperation with SmVKR1 we functionally analyzed this molecule in more detail. RNAi-mediated SmILK knock-down experiments were performed with *S*. *mansoni* couples *in vitro*, and the knock-down value determined by qPCR to be nearly 90% (S5 Fig). Following treatment with SmILK-dsRNA, pairing stability was not affected, and the amount of couples was similar to the untreated control group. However, egg production per (remaining) couple of the treated group significantly decreased during the observation period from 48 h post treatment on compared to the control (Fig 6).

**Fig 7:**
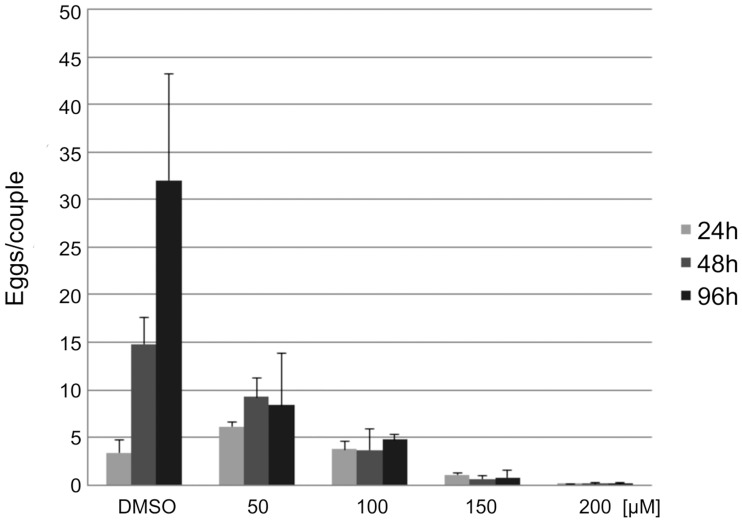
Egg production of worm couples decreases following QLT-0276 treatment *in vitro*. Analyses of egg production of adult *S*. *mansoni in vitro* following inhibitor treatment with the ILK inhibitor QLT-0276, which was used in different concentrations as indicated. Egg production significantly decreased from 48 h post treatment on in a concentration-dependent manner.

**Fig 8:**
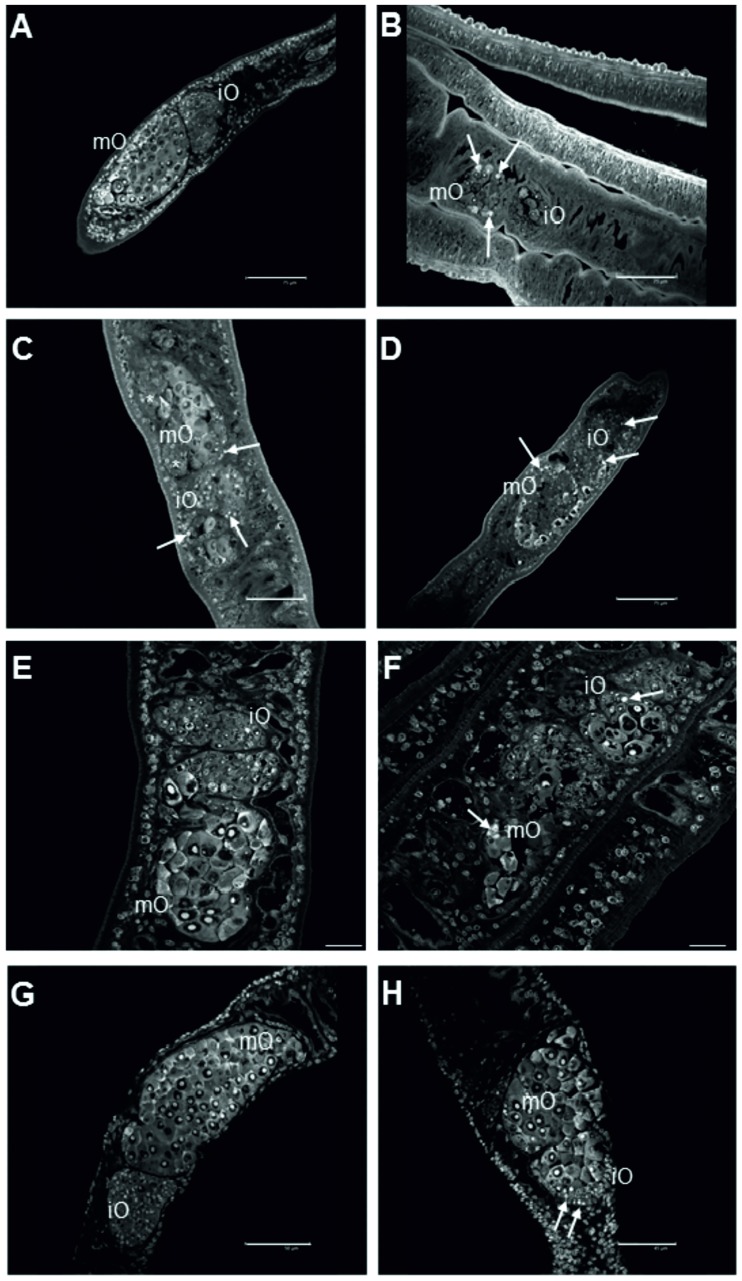
Morphological analyses show corresponding effects of QLT-0276 and RNAi in the ovary of female worms. CLSM analyses of paired females ((A) DMSO-treated female as control) under the influence of the inhibitor QLT-0276 (treatment for 3 days (B-D) or 14 days (E, F)) or ILK ds-RNA (25 μg, 4 days (G, H)) with a focus on the ovary. The ovary is a bulb-like structure with a smaller anterior part containing immature oogonia (iO) and a bigger posterior part containing mature primary oocytes (mO). As shown before [14, 15] the structure of the ovary and the integrity of oocytes were not negatively influenced by DMSO ((A) 3-day treatment). However, following treatment with 50 μM (B), 100 μM (C) or 200 μM (D) QLT-0276, mO appeared within the anterior part of the ovary (B, C) and iO in its posterior part (C, stars). Additionally, signs of oocyte degradation were observed in both parts of the ovary (arrows). After 14 days of *in vitro* culture the structure of the ovary of DMSO-treated females was unchanged (E) whereas ovaries of females treated with 10 μM QLT-0276 showed the deleterious phenotype (F) described above. In contrast to a control using GFP-dsRNA (G), in ILK dsRNA-treated paired females, the number of mO was smaller, they also appeared within the anterior part of the ovary (H), and iO in its posterior part, and signs of degeneration were observed, too (H). Scale bars are 75 μm (A, B, D), 50 μm (C, E), 45 μm (H) or 25 μm (E, F).

Inhibiting ILK was also achieved by QLT-0267, and following treatment with different concentrations (50–200 μM) a negative effect on pairing stability was observed. Furthermore, also egg production per remaining couple decreased in a concentration-dependent manner from 48 h post treatment on (Fig 7). Morphologically, CLSM analysis showed effects of QLT-0267 on oogenesis in paired females. This inhibitor caused not only a reduction of oocyte number and the mislocalization of oocytes of various stages of differentiation in the different parts of the ovary but also oocyte degeneration (Fig 8). The intensity of the phenotype increased with QLT-0276 concentration. A similar oocyte-related observation was made by RNAi in ILK-dsRNA treated paired females (Fig 8), although the strength of the observed phenotype (less oocytes, mislocalization, degeneration) was weaker compared to inhibitor treatment.

### Evidence for apoptosis in the ovary of paired females following SmILK impairment by QLT-0276 or RNAi

**Fig 9:**
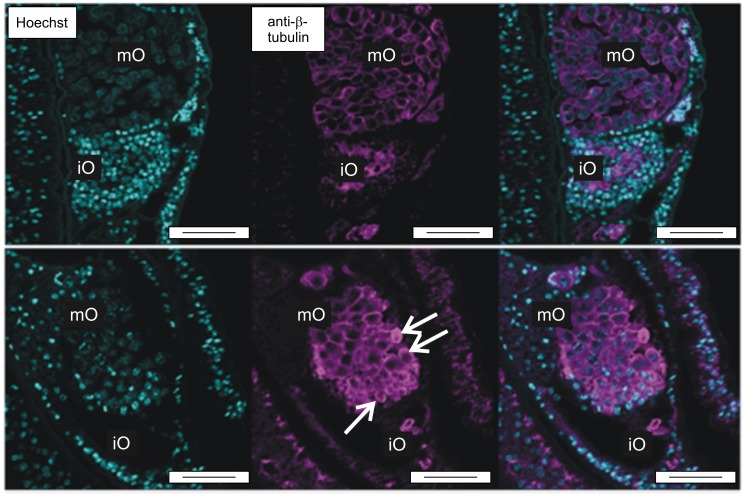
QLT-0276 reduces oocyte numbers and affects their morphology in paired females. Fluorescence microscopy of paired females with a focus on their ovaries. Females were treated for 3 days with DMSO (upper panel) or QLT-0276 (100 μM, lower panel) before staining with Hoechst 33342 (left) or anti-β-tubulin (middle). Merged images are shown right. Arrows indicate compact and rounded primary oocytes within the QLT-0276-treated group. iO, immature oogonia; mO, mature primary oocytes; scale bars: 50 μm.

Previous studies in cancer cells provided evidence that among other functions ILK is involved in cytoskeletal reorganization and cell survival, and its deregulation can contribute to errors in cell division and genomic instability [48]. Microtubule disruption was shown to induce cytoskeleton as well as cell adhesion changes. This led to focal adhesion kinase hydrolysis and the onset of apoptosis, a phenotype that was rescued by ILK overexpression [49]. Because there is evidence that apoptosis has a biological function for the maintenance of the maturation state of the reproductive organs of paired females [50], we investigated whether SmILK may be involved in this processes in *S*. *mansoni*. To this end we compared paired females treated with QLT-0267 and DMSO as control and performed immunolocalization with a β-tubulin antibody (S6 Fig). Under inhibitor influence the number of immature and primary oocytes was reduced in inhibitor-treated females. Compared to the control, primary oocytes clustered closer together, they appeared more compact, and some appeared as rounded up (Fig 9).

**Fig 10:**
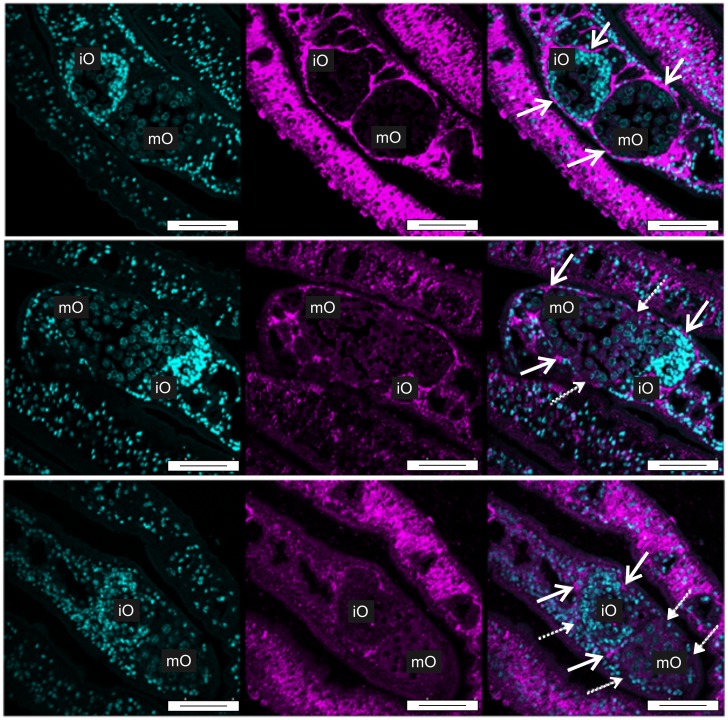
QLT-0276 affects the epithelium surrounding the ovary in paired females. Fluorescence microscopy of paired females with a focus on their ovaries. Females were treated for 3 days with DMSO (upper panel) or QLT-0276 (10 μM, middle panel; 100 μM, lower panel) before staining with Hoechst 33342 (left) or anti-α-laminin (middle). Merged images are shown right. Arrows indicate the α-laminin containing epithelium (upper) surrounding the ovary. In ovaries of treated females α-laminin staining was remarkably reduced or missing (dotted arrows). iO, immature oogonia; mO, mature primary oocytes; scale bars: 50 μm.

**Fig 11:**
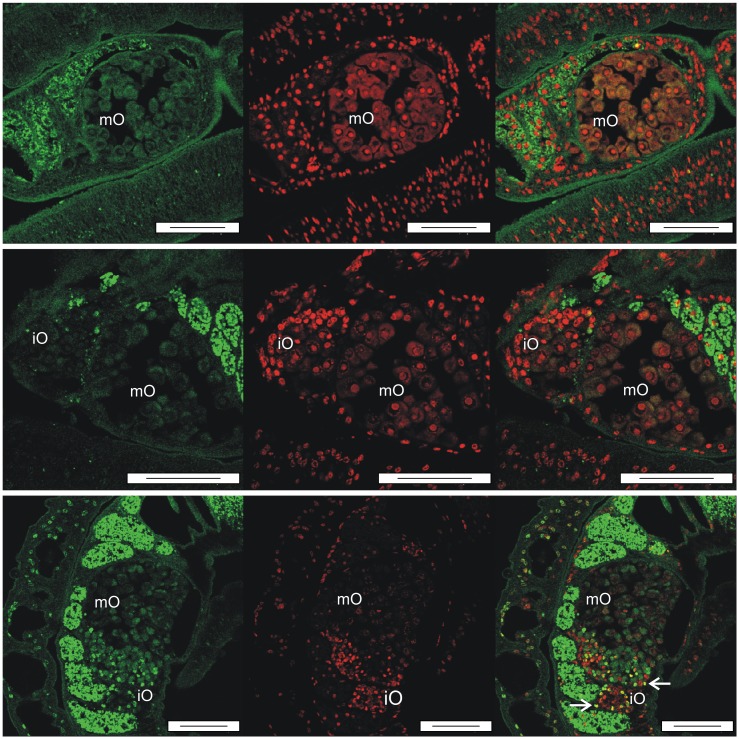
TUNEL-positive cells indicate apoptosis in oocytes of females treated with QLT-0276. Fluorescence microscopy to visualize the results of TUNEL assays performed with paired females treated for 3 days with DMSO (upper panel) or QLT-0276 (10 μM, middle panel; 100 μM, lower panel). Staining was performed by FITC-labeled dUTP (left) or propium iodide (middle). Merged images are shown right. Arrows indicate TUNEL-positive cells. iO, immature oogonia; mO, mature primary oocytes; scale bars: 50 μm.

**Fig 12:**
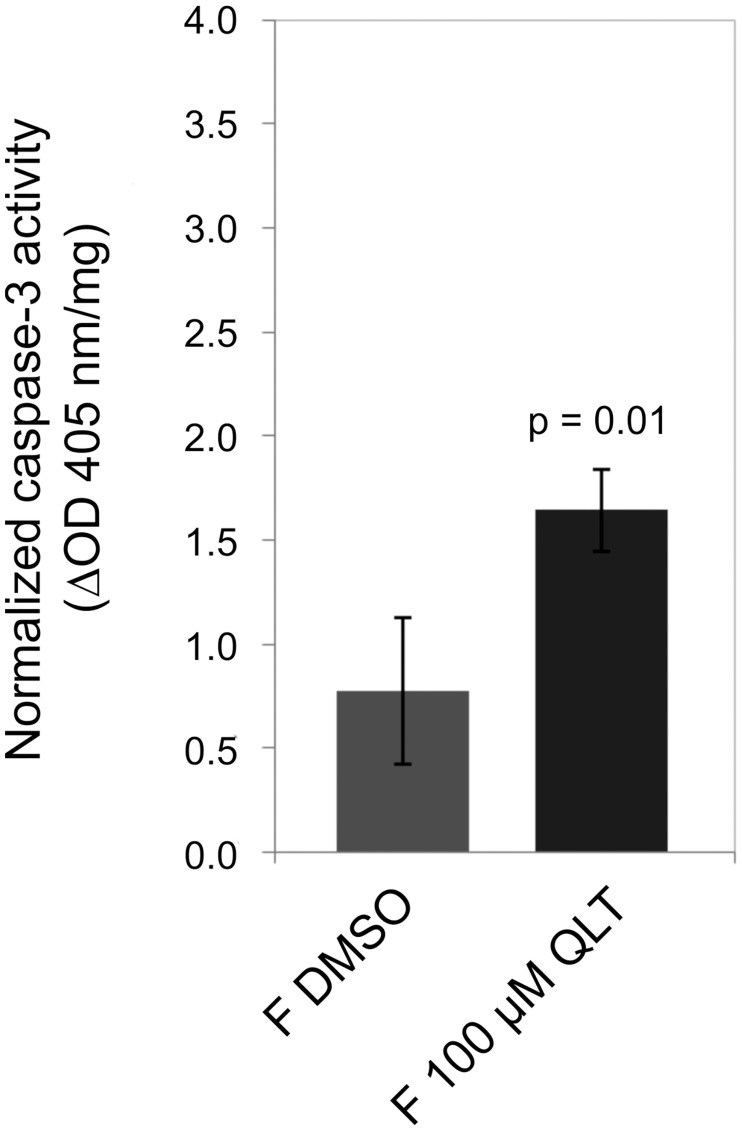
Caspase-3 activity increases following QLT-0276 treatment in paired females. Result of the caspase-3 activity assay showing that compared to a DMSO-treated control (left) the activity of caspase-3 of *S*. *man*soni increased significantly (p = 0.01) in paired females treated with 100 μM QLT-0276 (right) as determined spectrophotometrically (ΔOD_405_).

In a previous study first hints were obtained that laminins as extracellular matrix proteins may interact with Smβ-Int1 [17]. To investigate whether there is also an influence on components of the extracellular matrix we immunolocalized laminin in paired females treated with QLT-0267 or DMSO (S6 Fig). Indeed, a concentration-dependent decrease of laminin staining was observed within the epithelium surrounding the ovary of treated females (Fig 10). TUNEL assays finally confirmed apoptotic processes in ovaries of females treated with QLT-0267. TUNEL-positive cells occurred mainly within the smaller part of the ovary containing immature oocytes (Fig 11). To get further support for apoptotic processes induced by QLT-0267 in females, caspase-3 activity was determined in inhibitor-treated females. Following treatment the level of caspase-3 activity increased significantly (Fig 12). Next we investigated whether the expression of genes involved in early steps of apoptosis is affected. To induce the mitochondrial apoptosis pathway, a number of pro-apoptotic BCL-2 (B cell lymphoma 2) proteins collaborate with the outer mitochondrial membrane to permeabilize it. BAK (BCL-2 Antagonist Killer 1) and BAX (BCL-2 Associated X protein) are pro-apoptotic BCL-2 family members which are essential for the permebilization of the mitochondrial outer membrane [51]. We selected these genes because presumptive orthologs exist in the genome of *S*. *mansoni* (BAK, Smp_095190; BAX, Smp_072180). Therefore, we investigated the transcript profiles of Smp_095190 and Smp_072180 in schistosome females after treatment of couples for 72 h with 50 μM QLT. Compared to a DMSO control, we detected an upregulation of BAX after treatment, whereas BAK transcription remained constant. Upon RNAi both schistosome orthologs BAK and BAX were transcribed at higher levels (S7 Fig).

## Discussion

Although much research has been performed on integrins and integrin signaling in different organisms, there is not much known about their roles in platyhelminths. Here we report on an integrin-signaling complex in *S*. *mansoni* consisting of Smβ-Int1, SmILK, SmPINCH, SmNck2, and SmVKR1. According to phylogenetic analyses, SmILK, SmPINCH, SmNck2 form clusters that are specific for parasitic platyhelminths as it was shown for integrins before [17]. Together with the exclusive role of VKRs [25], it appears likely that parasites have modified the function of insulin-like signaling as well as integrins and their interacting partners for specific signaling purposes. Among these, at least one deals with the reproductive biology of platyhelminths. In this context schistosomes exhibit remarkable features because of the pairing-dependent development and maintenance of the differentiation status of female gonads. The involvement of schistosome VKRs and integrins for this physiological process has already been demonstrated [17, 21], and studies on the VKR ortholog AaeVKR of *A*. *aegypti* support the assumption of a specific role of VKRs for oogenesis and/or egg formation [23].

Our study provides first evidence for cooperation between integrin and VKR signaling in *S*. *mansoni*. This interaction is mediated by SmILK, SmPINCH, and SmNck2, cytoplasmic molecules with bridging function. Their colocalization with Smβ-Int1 and SmVKR1, especially in the ovary, indicated potential functions for the reproductive biology of schistosomes. In all three cases the intensities of localization signals in the ovary were higher in its posterior part which contains mature primary oocytes. Smβ-Int1 and Smα-Int1 were localized in the ovary—also dominating in its posterior part -, the vitellarium, the testes, the ootype-surrounding area, the subtegument, and within the parenchyma [17]. SmVKR1 expression was localized mainly in the female ovary, especially in mature, primary oocytes in the posterior part. In addition, SmVKR1 was also localized around the ootype and in the parenchyma of males [21]. Thus SmILK, SmPINCH, SmNck2 colocalized widely with Smβ-Int1 and SmVKR1 including their preferential occurrence in mature, primary oocytes, a prerequisite for potential interactions.

Different experiments with *Xenopus* oocytes expressing the complex members alone or in defined combinations of wildtype or mutated forms finally confirmed also by co-immunoprecipitation that a Smβ-Int1-SmILK-SmPINCH-SmNck2-SmVKR1 complex can be formed. GVBD assays demonstrated the biochemical function of this complex and the potentiality of SmVKR1 to be activated inside of the complex and to induce—in the absence of its ligand—processes leading to GVBD, which was confirmed by the results obtained. This suggests a new mode of SmVKR1 activation, which is achieved in a ligand-independent fashion by indirect cooperation with a β-integrin receptor. As mediators, GFR-specific and β integrin-specific adapter molecules operate, in this case SmILK, SmPINCH, and SmNck2. The participation of SmNck2 was shown indirectly by the use of a deletion mutant that negatively influenced GVBD, by Western blot analysis confirming the presence of *Xenopus* Nck2 in complexes without SmNck2, and finally by co-immunoprecipitation of SmNck2 after its addition.

In *Xenopus* oocytes, ligand-activated RTKs as IRs trigger the activation of Erk MAPK and PI3K/Akt/mTOR and JNK pathways resulting in meiotic maturation [52]. As shown for L-Arg-activated SmVKR1 [21], in our actual study the phosphorylation of Erk1/2, Akt, and JNK in *Xenopus* oocytes was achieved also by Smβ-Int1/SmVKR1 complex formation without ligand activation. Thus similar to IR activation, complex-activated SmVKR1 induced signaling processes involved in protein synthesis and cellular growth associated with *Xenopus* oocyte maturation, which substantiates the IR-like function of SmVKR1 but also its conjunction with oogenesis.

**Fig 13:**
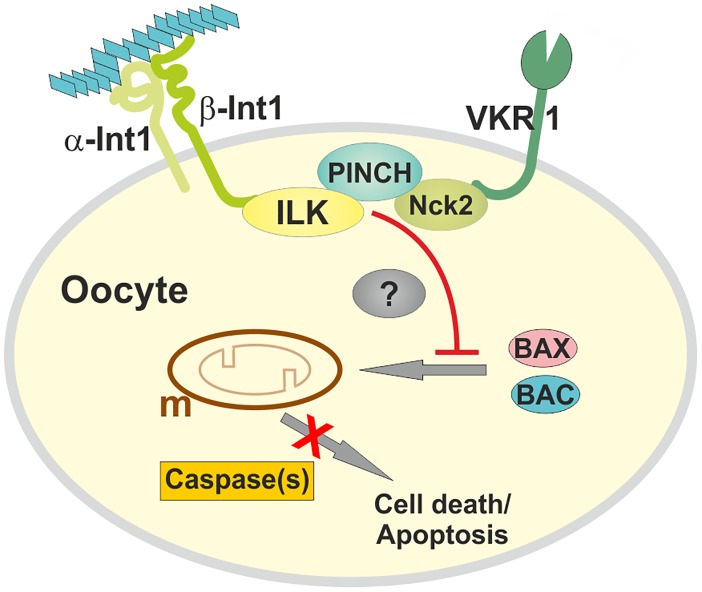
Hypothetical model of the function of the Smβ-Int1/SmVKR1 complex in mature *S*. *mansoni* oocytes. In paired *S*. *mansoni* females the complex formation of Smβ-Int1-SmILK-SmPINCH-SmNck2-SmVKR1 prevents cell death processes such as apoptosis mediated by the pro-apoptotic BCL-2 family members BAK and BAX and by caspases (red line, red cross). The process is probably induced by interaction of integrins (Smα/Smβ integrins) with the extracelluar matrix (blue trapezoids). Molecules mediating this effect from the membrane-located complex members to the cytosol are still unknown (?). m, mitochondrium.

Functional analyses of SmILK in *Xenopus* oocytes or as a member of the complex by RNAi and inhibitor studies finally indicated that this molecule represents a pseudokinase being involved in different processes in schistosomes. Among these is inside-out signaling in the ovary because the extracellular matrix as part of the epithelium surrounding the ovary was changed upon inhibiting SmILK as shown by laminin immunolocalization. Furthermore, SmILK appeared to control oocyte localization within the ovary, and oocyte survival. Inhibiting SmILK activity led to the reduction of the amount of immature oocytes and the degeneration of mature, primary oocytes. This is in part explained by apoptotic processes, for which evidence was obtained by TUNEL assays in case of immature oocytes, by determining caspase-3 activity which increased following inhibitor treatment, and by transcriptional analysis of the schistosome orthologs of BAK and BAX, two pro-apoptotic genes [51]. A recently conducted RNA-seq study revealed that both genes were expressed in schistosome females and within the ovary. Interestingly, the profiling of transcript abundance revealed that both genes were more abundantly transcribed in the ovaries of unpaired, immature females. After pairing, transcript abundance of both genes decreased in the ovary ([53]; S7 Fig). This supports the conclusion that apoptosis plays a role in oocyte differentiation, and that males exert a regulatory influence on this—suppressing apoptosis in the gonads of their female partners during a constant pairing contact (Fig 13). Degenerated primary oocytes were also detected that did not respond to TUNEL staining. Thus it seems feasible that further, apoptosis-independent processes leading to cell death contribute to oocyte degeneration.

In summary, the results presented here strongly suggest that the Smβ-Int1/SmVKR1 complex in the ovary of paired schistosomes is important for the maintenance of the differentiation status of oocytes and their survival. Against the background of the unusual reproductive biology of schistosomes this conclusion supports findings of a previous, independent study showing that apoptosis is used to control vitelline cell survival in a pairing-dependent manner in *S*. *mansoni* [50]. In this context our results match to a scenario of cell death processes controlling gonad maintenance in schistosome females and thus contribute to the understanding of biological processes controlling reproductive biology in this exceptional parasite. It has been hypothesized before that SmVKR1, possibly activated by L-Arg delivered with the male seminal fluid [21], is responsible for meiosis resumption and/or oocyte migration in schistosome females. In view of the new results it appears feasible that integrin-signaling contributes to this process providing a SmVKR ligand-independent alternative for activation (Fig 13). This could be achieved by mechanosensory forces. Indeed, integrins have been shown to sense, sort, and transduce mechanical forces into cellular responses. This form of integrin-based mechanotransduction contributes among others to cell growth, cell migration, gene expression including the activation of kinases, but also to apoptosis [54–57].

## Materials and Methods

### Ethics statement

Animal experiments using Syrian hamsters (*Mesocricetus auratus*) as model hosts were performed in accordance with the European Convention for the Protection of Vertebrate Animals used for experimental and other scientific purposes (ETS No 123; revised Appendix A) and were approved by the Regional Council (Regierungspraesidium) Giessen (V54-19 c 20/15 c GI 18/10).

### *Schistosoma mansoni* recovery and *in vitro*-culture experiments

*S*. *mansoni* was maintained in *Biomphalaria glabrata* as the intermediate host, with Syrian hamsters (*Mesocricetus auratus*) as the definitive hosts [36]. Adult worms were obtained by hepatoportal perfusion at day 46 or day 67 (in case of single sex infection; [58]) post-infection, respectively, and kept in M199 medium (Gibco) supplemented with 10% newborn calf serum and 1% ABAM-solution (10,000 units penicillin, 10 mg streptomycin and 25 mg amphotericin B per ml) at 37°C and 5% CO_2_ for 24h until the experiments started.

Couples were cultivated in 6-well plates in groups of eight per well (*n = 3*) and 3 ml supplemented M199 medium for RNA interference (RNAi) experiments (see below) or inhibitor studies. The latter were performed with the integrin-linked kinase (ILK) inhibitor QLT-0267 (Dermira, Inc., USA; [59]) which was added at final concentrations and period of times as indicated. It targets the ATP-binding site of ILK and was shown to be as effective as siRNA-mediated depletion of ILK [60]. Since ILK exerts no catalytic function, the inhibitory effect of QLT-0267 was explained by an impairment of the stability of ILK [61]. Equivalent volumes of dissolvent DMSO was used as control. Worms were monitored by bright-field microscopy (CX21, Olympus; Labovert FS, Leitz) over periods of 24 h– 96 h to analyze pairing stability, egg production, gut peristalsis and movement.

### Cloning and sequence analysis

For cloning of the full-length cDNAs of SmILK (Smp_079760), SmPINCH (Smp_020540.2), and SmNck2 (Smp_014850), total RNA was isolated from adult schistosomes using Trizol reagent (Invitrogen). Residual DNA was removed by DNase digestion (RNAeasy kit, Qiagen) following the manufacturer’s instruction. RNA quality was checked by Bioanalyzer microfluidic electrophoresis (Agilent Technologies). Starting RT-PCR the synthesis of cDNA was performed with 1 μg RNA using QuantiTect Reverse Transcription Kit (Qiagen). PCR reactions were performed in a final volume of 25 μl using primer end concentrations of 800 nM, denaturation at 95°C for 30 sec, annealing at 54°–64°C depending on the primer combinations (S1 Table), and elongation at 72°C for up to 2 min, and using FirePol-Taq (Solis biodyne). As vectors for cloning, pACT2 (Clontech), pcDNA3.1 (Invitrogen), or pBridge (Clontech) were used for directional cloning via restriction enzyme sites. Full-length SmILK cDNA was cloned via *Not*I and *Xba*I into pcDNA3.1, full-length SmPINCH cDNA via *Eco*RI/*Pst*I into pBridge, and full-length SmNck2 via *Bam*HI and *Xba*I into pcDNA3.1 (S1 Table). Primers designed for RT-PCRs to generate these cDNAs contained appropriate restriction sites for cloning. The sequence integrities of all cloned cDNAs were verified by sequencing (LGC Genomics, Berlin).

### Gonad isolation

Ovaries of female worms were isolated using the combined detergents/enzyme-based organ isolation protocol [42]. In short, isolated adult females (about 50 each) were transferred into 2 ml-reaction vessels and washed twice with 2 ml of non-supplemented M199-medium at room temperature. The medium was removed, and 500 μl of tegument solubilisation (TS)-solution was added (0.1% of each following compounds in DEPC (diethylpyrocarbonate)/PBS (phosphate-buffered saline): Brij 35 (Roth), Nonidet P40-Substrate (Fluka), Tween80 (Sigma) and TritonX-405 (Sigma), pH 7.2–7.4) followed by incubation in a thermal shaker (TS-100, Biosan) for 5 min at 1,200 rpm at 37°C. Shaking was repeated twice, and the solution was replaced after each cycle. Then the worms were rinsed three times with M199 and subsequently treated with elastase (300 μl elastase solution: 5 U/ml in M199; Sigma) at 37°C and 650 rpm in the thermal shaker to release the ovaries. Digestion was monitored by bright-field microcopy (Leica) and stopped when the gonads were released from the disrupted and digested worm carcasses. Finally, the gonads were manually collected by pipetting and transferred into supplemented M199 medium.

### *In situ*-hybridization

Sample preparation of *S*. *mansoni* adults was conducted as described previously [62]. In short, schistosome pairs were fixed in Bouin's solution (picric acid/acetic acid/formaldehyde; 15/1/5) followed by embedding in paraplast (Paraplast plus, Sigma). Sections of 5 μm thickness were incubated in xylol and after rehydration, the sections were treated with proteinase K (1 μg/ml) and dehydrated. As probe, *in vitro*-generated transcripts were synthesized and labeled with digoxigenin as suggested by the manufacturer (Roche). The correct sizes of labeled sense and antisense transcripts were checked by gel electrophoresis, and the RNA quality was tested by blotting and detection of digoxigenin using alkaline phosphatase-conjugated anti-digoxigenin antibodies, naphtol-AS-phosphatase, and Fast Red TR (Sigma). *In situ*-hybridization was performed at 57°C for 16 h. Afterwards, the sections were washed up to 0.5 × SSC (75 mM NaCl, 7.5 mM sodium citrate, pH 7.0), and detection of alkaline phosphatase was performed as mentioned above.

### Co-immunoprecipitation following expression in *Xenopus* oocytes and Western blot analyses

The intracellular part of Smβ-Int1 containing the C-terminus with an HA-tag at its N-terminus was subcloned into pcDNA 3.1 (Invitrogen) as described earlier [17]. Capped messenger RNA (cRNA) encoding Smβ-Int1 C-term was synthesized *in vitro* (T7 mMessage machine Kit, Ambion, USA) following a previously established protocol [45]. Furthermore, V5-tagged SmILK and SmPINCH and Flag-tagged SmNck2 were cloned the same way into pcDNA 3.1, and their sequence identities confirmed by commercial sequencing. Also cRNAs were prepared from these clones as well as from V5-tagged SmVKR1 variants (wildtype SmVKR^wt^, dead kinase mutant SmVKR^dk^ [= KO], and a constitutively active mutant SmVKR1^YYRE^ [= XE]) cloned in pcDNA 3.1 as reported in a previous study [22]. Interaction studies between these proteins (see results) were done by co-injecting different cRNA combinations into *Xenopus* oocytes as reported before [17, 45]. Expressed proteins were detected by immunoprecipitation and Western blot analyses. Following the standard procedure [45], 30 oocytes were lysed in 300 μl of buffer (50 mM HEPES, pH 7.4, 500 mM NaCl, 5 mM MgCl2, 1 mg/ml bovine serum albumin, 10 μg/ml leupeptin, 10 μg/ml aprotinin, 10 μg/ml soybean trypsin inhibitor, 10 μg/ml benzamidine, 1 mM PMSF, 1 mM sodium vanadate) after 5 h or 15 h of expression. Following centrifugation at 4°C for 15 min and 10,000 g, the resulting supernatants were incubated with anti-HA (1:100; Invitrogen) or anti-V5 (1:100; Invitrogen) then added to protein A-Sepharose beads (5 mg, Amersham Biosciences) for 1 h at 4°C. After washing three times, immune complexes were eluted from the beads in Laemmli buffer and analyzed by SDS-PAGE (7.5%–15% polyacrylamide gels).

Western blot analyses were performed using anti-V5 (1: 50,000), anti HA (1: 50,000), anti-Flag (1: 1,000), anti-human nck2 (1: 1,000, nck2(8.8): sc-20020, Santa Cruz Biotechnology), or PY20 (1: 10,000; anti-phosphotyrosine, BD Biosciences) antibodies. The following primary antibodies were applied to confirm the presence of total or phosphorylated ERK2, JNK and Akt kinases: anti-ERK2 (1: 10,000; Santa Cruz Biotechnology), anti-phospho p44/p42 MAPK (ERK1/2; Thr 202/Tyr 204; 1: 10,000; Cell Signalling Technology), anti-c-jun N-terminal kinase JNK (1: 10,000; Sigma), anti-active JNK polyclonal antibody (1: 8,000; Promega), anti-Akt1 (C-20; 1: 5,000; Santa Cruz Biotechnology), anti-phospho Akt (Thr308; 1: 5,000; Upstate Biotechnology) and anti-phospho Akt (Ser 473; 1: 5,000; Upstate Biotechnology). Mouse, rabbit or goat Trueblot secondary antibodies (eBioscience) were used as secondary antibodies and chemoluminescence was detected using the advanced ECL detection system (Amersham Biosciences).

### RNA interference

Following standard protocols for RNAi in adult schistosomes [15, 63], double-stranded RNA of approximately 500 bp was synthesized (nucleotide position 559–1016) using the MEGAscript RNAi kit (Life Technologies). Gel electrophoresis in 1.2% agarose-MOPS was conducted to prove for single RNA bands of the correct size. Schistosome couples in groups of eight pairs (*n = 3*) were electroporated in the presence of 25 μg dsRNA and subsequently soaked *in vitro* for 96 h. Every 24 h the treated worms were inspected and different parameters evaluated such as pairing stability, egg production, gut peristalsis and movement.

### Qualitative and quantitative reverse transcription PCR

Schistosome samples were collected and transferred into PeqGOLD TriFast (Peqlab). After storage at -80°C or immediately after transfer, RNA isolation was done following the manufacturer’s instructions (Peqlab). About 500 ng total RNA was used for cDNA synthesis using the Quantitect Reverse Transcription Kit (Qiagen). For PCR, 1 μl of a 1:20 dilution of cDNA was tested using exon-spanning PDI (protein dilsufide isomerase) 5’/3’ primers; forward: 5´-AAATGATGCCCCGACTTACC-3´ and reverse: 5´- TCATCCCAAACTGGAGCAAG-3`[62, 64]) to confirm that the genomic DNA was properly removed. For quantitative RT PCR (qRT-PCR), a RotorGene-Q PCR cycler (Qiagen) was used and all reactions were set up in triplicates. Each reaction had a final volume of 25 μl; 5 μl of a 1:20 cDNA served as template and 125 nM (final concentration) of each primer were added to 12.5 μl of 2x PerfeCTa SYBR Green super mix (Quanta). No template controls (NTC) were included in each run. RNAi-mediated knockdown of gene expression was analysed by absolute quantification. Therefore, a standard curve on diluted gel eluate was included in each run [65]. For qPCR analyses to study transcript profiles of SmBAK (Smp_095190), SmBAX (Smp_072180), SmmTor (Smp_122910), and SmSod (Smp_056440) several genes were tested for their suitability as reference. Based on transcriptome data [53] the gene Smp_008900 (annotated as eukaryotic translation initiation factor 4 gamma) fulfilled this criterion and was further tested under various condition using an absolute quantification approach. To this end, the Smp_008900 amplicon was cloned into pDrive (Qiagen) and served as template in dilution series. Different cDNAs of electroporated and inhibitor-treated *S*. *mansoni* couples confirmed constant numbers of Smp_008900 transcripts (primers, see S1 Table).

### TdT-mediated dUTP nick end labeling (TUNEL) assay

Sliced specimens of 4 μm thickness on slides were deparaffinized, dehydrated and then equilibrated in proteinase-K buffer (100 mM Tris/Cl, 50 mM EDTA pH 8.0) for 5 min. Subsequently, treatment with proteinase K (1 μg/ml) was performed for 20 min at 37°C. Afterwards, slides were rinsed once with 1x PBS and immersed twice with 500 μl washing buffer provided by the fluorometric DNA-fragmentation detection kit III (F-dUTP; Promokine) for 5 min at ambient temperature. The staining solution containing FITC-labeled dUTP was prepared according to the manufacturer’s instruction (Promokine). A control solution was prepared without TdT enzyme. Subsequently, specimens were immersed with 100–200 μl of the staining solution and kept in the dark. Following incubation for 60 min at 37°C, the slides were rinsed twice with 500 μl rinse buffer (Promokine) for 5 min at ambient temperature and counterstained with 200 μl propidium iodide/RNase A solution (Promokine) for 15 min. Slides were finally mounted with FluoroMount (Roth) and analyzed within 3 hours after staining by fluorescence microscopy (Ex/Em = 488/520 nm for FITC, and 488/623 nm for PI).

### Caspase-3 activity assay

Following perfusion, *S*. *mansoni* couples were taken in culture and treated with DMSO or with QLT-0267 (100 μM) for 72 h. Following treatment the couples were carefully separated with featherweight forceps, and females and males transferred separately into 1.5 ml tubes with 500 μl 1x PBS for washing. After sedimentation, the supernatant was replaced by 50 μl cold (4°C) cell lysis buffer of the caspase-3 colorimetric assay kit (Promokine) and the samples kept on ice. After 10 min incubation, worms were homogenized with sterile pestils and kept on ice for further 10 min. Debris were subsequently sedimented by 10.000 x g centrifugation for 2 min at 4°C and afterwards placed back on ice. Subsequently, 20 μl of the supernatant were mixed with 30 μl of pre-transferred cell-lysis buffer in 96-well plates, and 50 μl of two-fold reaction buffer complemented with 10 μM DTT was added. Two hours incubation at 37°C allowed cleaving the p-nitroanilide-labeled substrate DEVD (Promokine) that was added at a final concentration of 200 μM. Samples were read at 405 nm with a Varioscan plate reader (Thermo Fisher Scientific). Samples were normalized to the protein concentration that was determined using the BCA assay kit (Pierce) promptly after the caspase-3 assay was set up. The results relied on the analysis of three biological replicates obtained from independent perfusions. In contrast to previously published protocols [50] caspase-3 activity was not detected when the homogenization step was omitted.

### Microscopic analysis

Adult worms were stained with carmine red for a general morphological analysis by CLSM according to previously published protocols [14, 66]. For microscopy (CLSM; Leica TSC SP2 microscope) and documentation the probes were excited with a 488 nm He/Ne laser and emission was captured with a 470 nm long-pass filter in reflection mode as described before [14].

Fluorescence microscopy of antibody-stained worm sections (5 μM) was done using an Olympus IX 81 inverted microscope. Anti-laminin (antibodies-online.com; LN, ABIN268409) and anti-β-tubulin (antibodies-online.com; anti-TUBB, ABIN269949) antibodies were used in concentrations of 1: 5,000 each, as recommended by the manufacturer (Novus Biologicals). As secondary antibody, a fluorescence-labeled goat anti-rabbit antibody was used (LI-COR Bioscience, IRDye 680LT; 1: 5,000). These antibodies were tested on lysates of adult schistosomes by Western blot analyses as described before [42] using 15 μg protein each, which had been size-separated by SDS-PAGE using 7.5%–15% polyacrylamide gels depending on the size of the protein to be detected.

### *In silico* analyses

The following public domain tools were used: BLASTx (http://www.ncbi.nlm.nih.gov/BLAST), SchistoDB (http://schistodb.net/schisto/; [67]), and WormBase ParaSite (release 6, April 2016; http://parasite.wormbase.org/; [37]). The online-tool SMART (http://smart.embl-heidelberg.de/) [68] was used to predict protein domains. Primer3Plus was used for primer design (http://www.bioinformatics.nl/cgi-bin/primer3plus/primer3plus.cgi) and Oligo Calc for analyzing primer properties (http://www.basic.northwestern.edu/biotools/oligocalc.html).

## Supporting Information

S1 Table List of primers used. (DOCX)

S1 Fig Structure and phylogenetic analyses of SmILK. (PDF)

S2 Fig Structure and phylogenetic analyses of SmPINCH. (PDF)

S3 Fig Structure and phylogenetic analyses of SmNck2. (PDF)

S4 Fig RT-PCR confirming transcripts of SmILK, SmPINCH, and SmNck2 in isolated gonads of *S*. *mansoni*. (PDF)

S5 Fig RNAi-mediated knockdown of SmILK. (PDF)

S6 Fig Western blot analyses to test the specificity of antibodies. (PDF)

S7 Fig Transcript profiles of the pro-apoptotic genes BAK and BAX of *S*. *mansoni*. (PDF)
