# Supplementary material for: Evidence for Integrin – Venus Kinase Receptor 1 Alliance in the Ovary of Schistosoma mansoni Females Controlling Cell Survival
Source: PLoS Pathog. 2017 Jan 23;13(1):e1006147. doi: 10.1371/journal.ppat.1006147 (PMC5289644; doi:10.1371/journal.ppat.1006147)
Supplement: S1 Table — (DOCX) [file ppat.1006147.s001.docx]

**Supplementary table S1: List of primers used**

| **Names of molecules** | **Purposes** | **Primer names** | **Primer sequences (5´-3´)** |
| --- | --- | --- | --- |
| SmILK | cloning | ILK-*Not*I-fw1  SmILK-2-fwd(*Nco*I) | *GCGGCCGC*ATGGAAACTATTATTGCTCATGTTC  *CCATGG*AAACTATTATTGCTCATGTTC |
|  |  | ILK-*Xba*I-rev  SmILK-2-rev(*Xma*I) | *TCTAGA*ATTGAGAAGCACGTTCACGC  *CCCGGG*GCTCATTGAGAAGCACGTTCAC |
| SmILK | deletion cloning | SmILK2-Del_ANK1-fwd(*Nco*I  SmILK-2-rev(*Xma*I) | *CCATGG*GTGATGACACAG  *CCCGGG*GCTCATTGAGAAGCACGTTCAC |
| SmILK | ish | ILK-fw4  ILK-rev5 | GAAGGTGAATCATGGGATCCTCG  GCAATATCTTCGTCCACCATTACG |
| SmILK | dsRNA  synthesis | ILK-T_7_-fw3  ILK-T_7_-rev4 | *TAATACGACTCACTATAGGGAGA*GAAGGTGAATCATGGGATCCTCG  *TAATACGACTCACTATAGGGAGA*GCAATATCTTCGTCCACCATTACG |
| SmILK | qPCR | qILK-fw  qILK-rev | GGAGCATGTCTTATGCAAG  CCAATCCAGCTTGTATCACG |
| SmPINCH | cloning | PINCH-*Eco*RI-fw  PI-pACT-fwd(*Nco*I)  Pinch-*Xho*I-rev  PI-pACT-rev(BamHI) | *GAATTC*ATGCTTCATGAGGCACG  *CCATGG*AGATGCTTCATGAGGCACG  *TGGAGT*AAGAGTTCCTGTCTTGA  *GGATCC*CTTAATTACTCCATTTTTGTCTATCTGA |
| SmPINCH | deletion cloning | Pinch-Del4Lim-fw  Pinch-Del4Lim-rev | GTCTGTCATATATGCGCGAAAC  TGATACACCAGAACGACTGAAAC |
| SmPINCH | ish | Pinch-si-fw | GTGATCAAGAACTTGGTTTGGATG |
|  |  | Pinch-si-rev | ATTGCATTGGTAACTTATCATAAC |
| SmPINCH | dsRNA  synthesis | Pinch-T_7_-fw | *TAATACGACTCACTATAGGGAGA*GTGATCAAGAACTTGGTTTGGATG |
|  |  | Pinch-T_7_-rev | *TAATACGACTCACTATAGGGAGA*ATTGCATTGGTAACTTATCATAAC |
| SmPINCH | qPCR | qPinch-fw  qPinch-rev | GTGGTGAAAGCAATGAATCGAAG  GTTTGACAGATATGACAGCCAAC |
| SmNck-2 | cloning | Nck2-*Bam*H1-fw1 | *GGATCC*ACATGATGTCTCATAAAAATACGG |
|  |  | Nck2-*Xba*1-rev1 | *TCTAGA*AGAAAAACGATTGTGTGGAGCAG |
| SmNck-2 | deletion cloning | NckDEL3SH3-fw  NckDEL3SH3-rev | ACACCACCGACGGCAACTACAAC  ATGTTGCTGTTGCTGACCTTCATG |
| SmNck-2 | ish/qPCR | Nck2-ds-fw  Nck2-ds-rev-2 | ACACTTAATCGGGATGTAAATG  CAAGCAACCTGTACTAGACTG |
| SmNck-2 | dsRNA  synthesis | Nck2-T_7_-fw  Nck2-T_7_-rev-2 | *TAATACGACTCACTATAGGGAGA*ACACTTAATCGGGATGTAAATG  *TAATACGACTCACTATAGGGAGA*CAAGCAACCTGTACTAGACTG |
| SmEIF4G1 | qPCR | qSm8900-fw  qSm8900-rev | ACCGCACACGCTGTTGATG  GCAACGGCAAGAACTTCGG |
| SmBAK | qPCR | qSmBak-fw  qSmBak-rev | GCCGACGAACTTCAACTGATATG  CTTCTCATATGTGGCTGACTTG |
| SmBAX | qPCR | qSmBax-fw  qSmBax-rev | CGATGAGAAGTTGAAGAATTGG  GCAAGCCCACCAACATTACG |
| SmmTOR | qPCR | qSmmTOR-fw  qSmmTOR-rev | GAACCACATGGCAATCTCGC  ACACAGTCCAGCAAAGCGTA |
| SmSod | qPCR | qSmSod2-fw  qSmSod2-rev | CAATTGCTGTCCAGGGTTCG  ATGCATGCTCCCAAACGTCG |
|  |  |  |  |

T_m_°C, annealing temperature; fw, forward; rev, reverse.; ish, *in situ* hybridization. Recognition sequences for restriction enzymes as parts of some the primer sequences are indicated by italics as well as sequences representing the T_7_ promoter. Appropriate restriction enzymes (or T_7_ promotor, respectively) are parts of the primer names given. SmEIF4G1, ortholog of the eukaryotic translation-initiation factor 4 gamma (Smp_008900), which was used as reference gene for qPCR.
