# Supplementary material for: Evidence for Integrin – Venus Kinase Receptor 1 Alliance in the Ovary of Schistosoma mansoni Females Controlling Cell Survival
Source: PLoS Pathog. 2017 Jan 23;13(1):e1006147. doi: 10.1371/journal.ppat.1006147 (PMC5289644; doi:10.1371/journal.ppat.1006147)
Supplement: S1 Fig — (PDF) [file ppat.1006147.s002.pdf]

Supplementary figure S1: Structure and phylogenetic analysis of SmILK

A

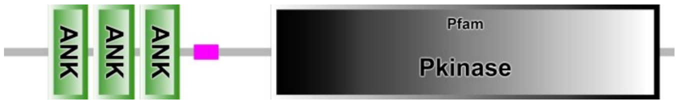

B

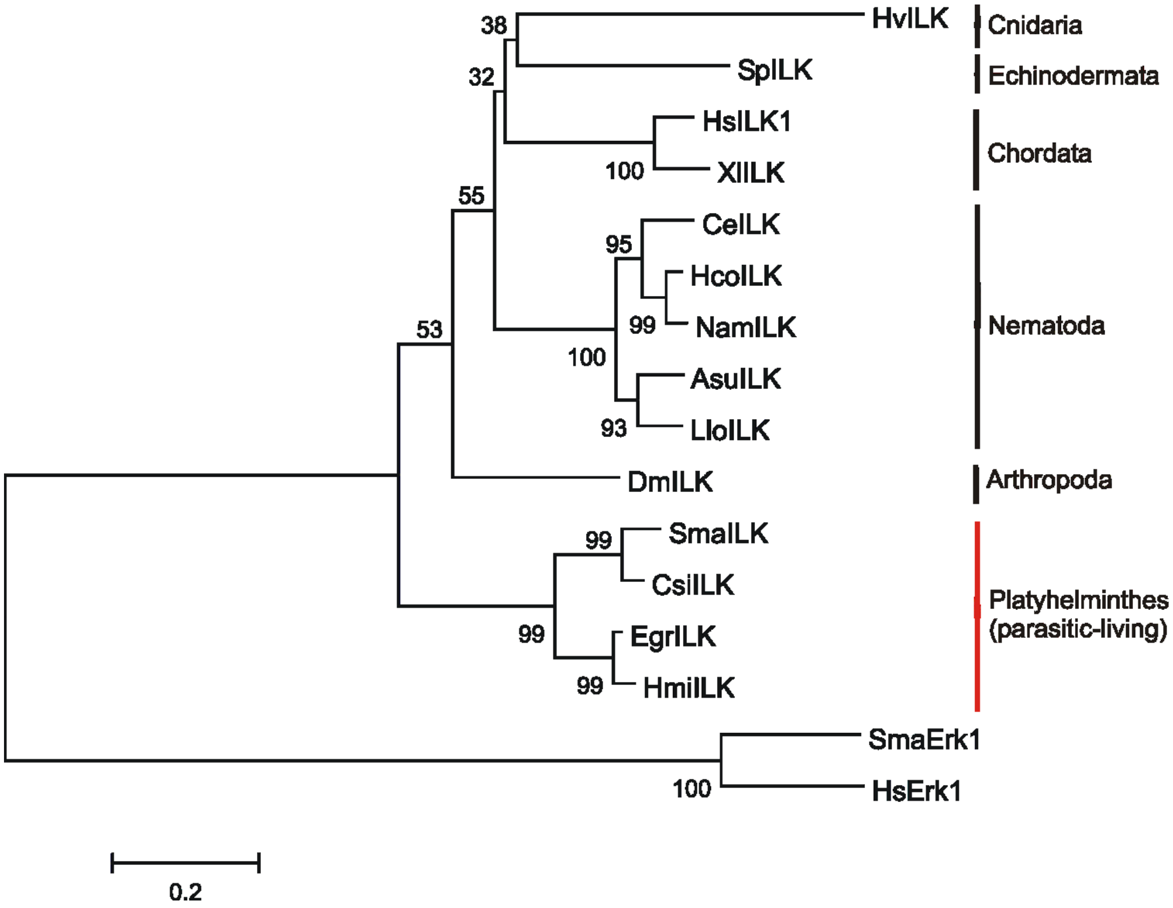

Suppl. fig. S1

A, Structure of SmILK (Smp_079760) analysed by SMART showing three ankyrin domains (ANK) and the pseudokinase-like domain (Pkinase).

B, Phylogenetic analysis using the Neighbor-Joining method [1-4]. In addition to SmILK (SmaILK) the analysis included the following 15 amino acid sequences of orthologs of organisms of different phylogenetic origins such as *Hydra vulgaris* (HvILK; Genbank accession number: XP_002158769), *Strongylocentrotus purpuratus* (SpILK; XP_786444), *Homo sapiens* (HsILK1; NP_004508), *Xenopus laevis* (XlILK; NP_001086805), *Caenorhabditis elegans* (CeILK; NP_497139), *Haemonchus contortus* (HcoILK; CDJ97346), *Necator americanus* (NamILK;

ETN76257), *Ascaris suum* (AsuILK; ERG84143), *Loa loa* (LloILK; XP_003136221), *Drosophila melanogaster* (DmILK; CAB77053), *Clonorchis sinensis* (CsiILK; GAA48258), *Echinococcus granulosus* (EgrILK; CDJ17537), *Hymenolepis microstoma* (HmiILK; CDS28855) and two amino acid sequences of the related serine/threonine kinases Erk of *Schistosoma mansoni* (SmaErk1;CCD60922) and *Homo sapiens* (HsErk1; NP_002737) serving as out-group. All positions containing gaps and missing data were eliminated. In Planaria, the free-living class of the phylum platyhelminthes, no homolog was found by BLAST. Parasitic living species names were abbreviated with the first two letters, free-living with the first.

References

1. Felsenstein J. Confidence limits on phylogenies: An approach using the bootstrap. Evolution 1985;39: 783-791.

2. Saitou N, Nei M. The neighbor-joining method: A new method for reconstructing phylogenetic trees. Mol Biol Evol. 1987;4: 406-425.

3. Tamura K, Stecher G, Peterson D, Filipski A, Kumar S. MEGA6: Molecular Evolutionary Genetics Analysis version 6.0. Mol Biol Evol. 2013;30: 2725-2729.

4. Zuckerkandl E, Pauling L. Evolutionary divergence and convergence in proteins. In: Bryson V, Vogel HJ, editors. Evolving Genes and Proteins. Academic Press, New York; 1965. pp. 97-166.
