# Supplementary material for: Evidence for Integrin – Venus Kinase Receptor 1 Alliance in the Ovary of Schistosoma mansoni Females Controlling Cell Survival
Source: PLoS Pathog. 2017 Jan 23;13(1):e1006147. doi: 10.1371/journal.ppat.1006147 (PMC5289644; doi:10.1371/journal.ppat.1006147)
Supplement: S2 Fig — (PDF) [file ppat.1006147.s003.pdf]

Supplementary figure S2: Structure and phylogenetic analysis of SmPINCH

A

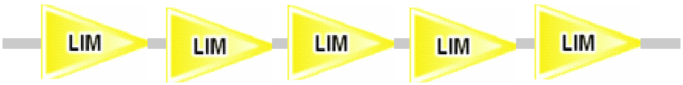

B

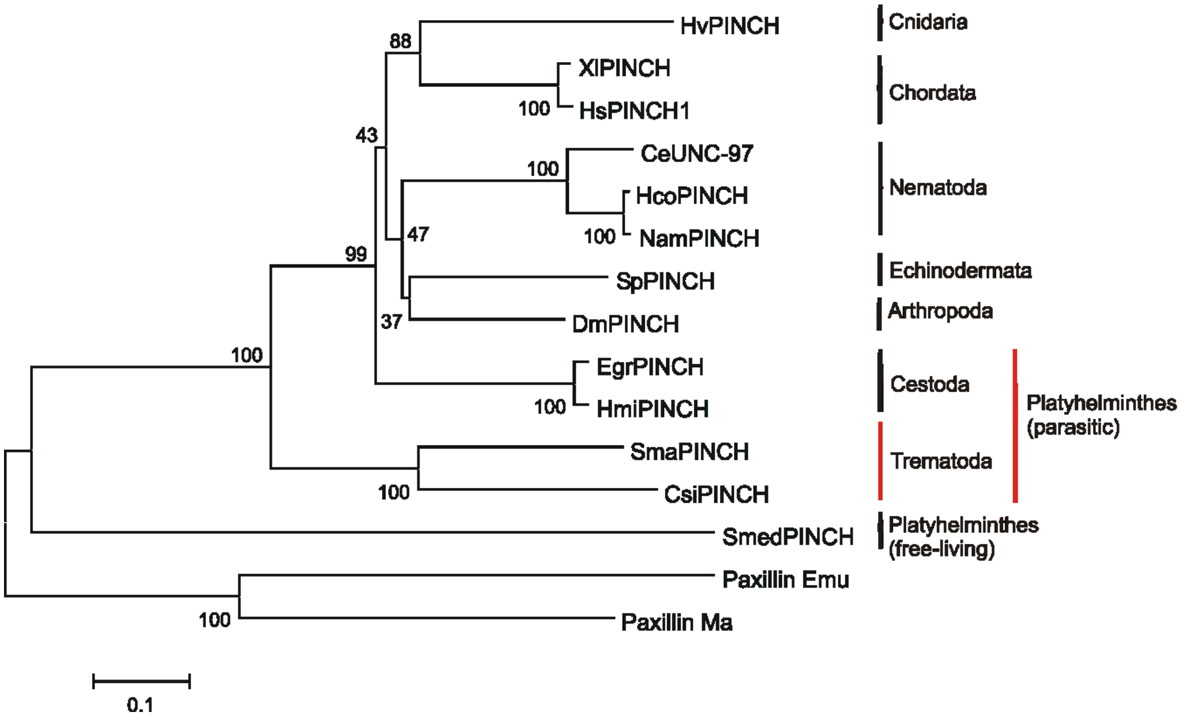

Suppl. fig. S2

A, Structure of SmPINCH (Smp_020540.2) analyzed by SMART showing five LIM domains.

B, Phylogenetic analysis using the Neighbor-Joining method [1-4]. In addition to SmPINCH (SmaPINCH) the analysis involved the following 14 amino acid sequences of orthologs of organisms of different phylogenetic origins such as *Hydra vulgaris* (HvPINCH; XP_002164622), *Xenopus laevis* (XlPINCH; ABS17667), *Homo sapiens* (HsPINCH; NP_001180413), *Caenorhabditis elegans* (CeUNC-97; CCD83467), *Haemonchus contortus* (HcoPINCH; CDJ80147), *Necator americanus* (NamPINCH; ETN83005), *Strongylocentrotus purpuratus* (SpPINCH; XP_793010), *Drosophila melanogaster* (DmPINCHs; NP_731242), *Echinococcus granulosus* (EgrPINCH; EUB60965), *Hymenolepis microstoma* (HmiPINCH; CDS30700), *Clonorchis sinensis* (CsiPINCH; GAA38790), *Schmidtea mediterranea* (SmedPINCH; v31.005368) and the two amino acid sequences coding for LIM domain containing proteins of *Echinococcus multilocularis* (Paxillin Emu; CDI97526) and *Mesocricetus auratus* (Paxillin Ma; XP_005079036) serving as out-group. All positions containing gaps and missing data were eliminated.

References

1. Felsenstein J. Confidence limits on phylogenies: An approach using the bootstrap. Evolution 1985;39: 783-791.

2. Saitou N, Nei M. The neighbor-joining method: A new method for reconstructing phylogenetic trees. Mol Biol Evol. 1987;4: 406-425.

3. Tamura K, Stecher G, Peterson D, Filipski A, Kumar S. MEGA6: Molecular Evolutionary Genetics Analysis version 6.0. Mol Biol Evol. 2013;30: 2725-2729.

4. Zuckerkandl E, Pauling L. Evolutionary divergence and convergence in proteins. In: Bryson V, Vogel HJ, editors. Evolving Genes and Proteins. Academic Press, New York; 1965. pp. 97-166.
