# Supplementary material for: Evidence for Integrin – Venus Kinase Receptor 1 Alliance in the Ovary of Schistosoma mansoni Females Controlling Cell Survival
Source: PLoS Pathog. 2017 Jan 23;13(1):e1006147. doi: 10.1371/journal.ppat.1006147 (PMC5289644; doi:10.1371/journal.ppat.1006147)
Supplement: S3 Fig — (PDF) [file ppat.1006147.s004.pdf]

Supplementary figure S3: Structure and phylogenetic analysis of SmNck2

A

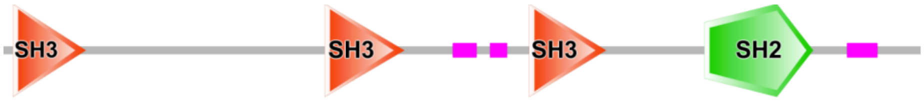

B

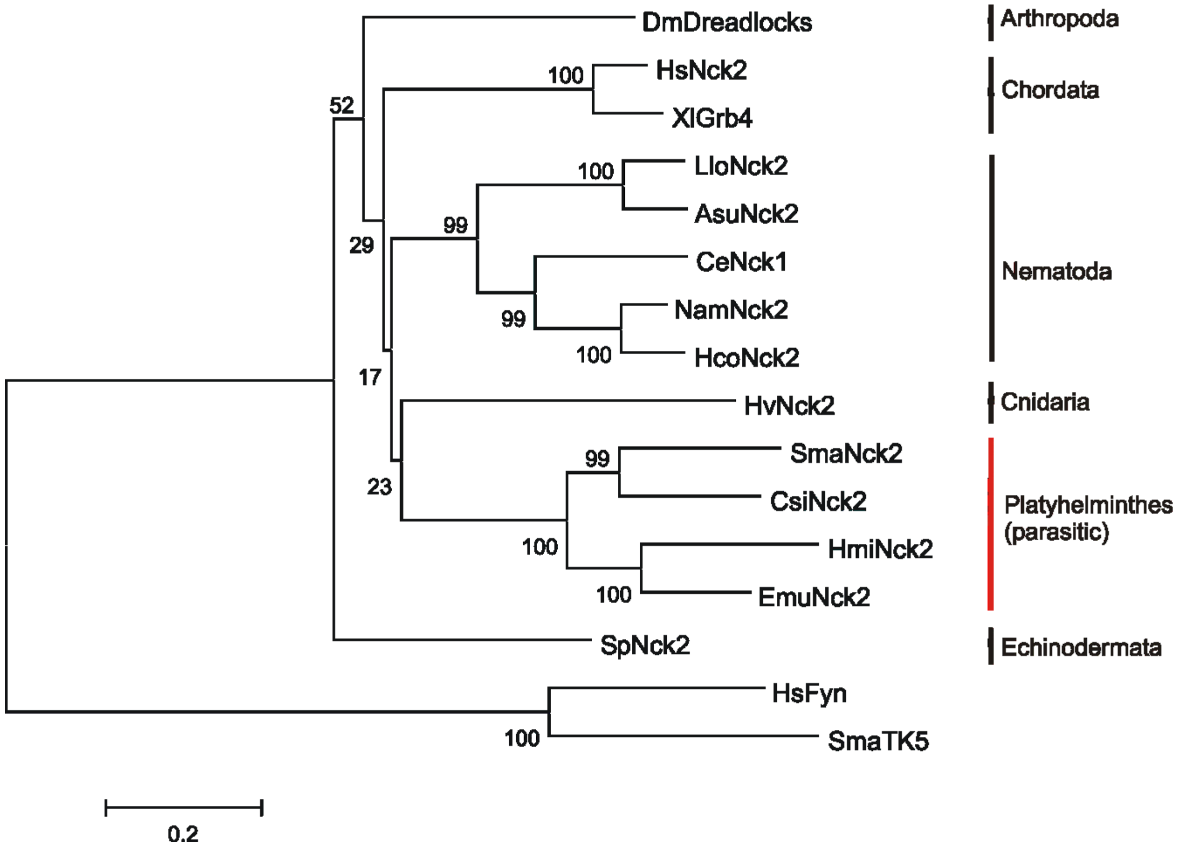

Suppl. fig. S3

A, Structure of SmNck2 (Smp_014850) analyzed by SMART showing three SH3 domains (SH3) and one SH2 (SH2) domain.

B, Phylogenetic analysis using the Neighbor-Joining method [1-4]. Besides SmNck2 (SmaNck2) the analysis involved the following 15 amino acid sequences of orthologs of organisms of different phylogenetic origins such as *Drosophila melanogaster* (DmDreadlocks; NP_722657), *Homo sapiens* (HsNck2; NP_003572), *Xenopus laevis* (XlNck2; NP_001083313), *Loa loa* (LloNck2; XP_003137411), *Ascaris suum* (AsuNck2; ERG86028), *Caenorhabditis elegans* (CeNck1; NP_508706), *Necator americanus* (NamNck2; ETN84925), *Haemonchus contortus* (HcoNck2; CDJ85790), *Hydra vulgaris* (HvNck2; XP_002160090), *Clonorchis sinensis* (CsiNck2; GAA50029), *Hymenolepis microstoma* (HmiNck2; CDS31037), *Echinococcus multilocularis* (EmuNck2; CDJ02918), *Strongylocentrotus purpuratus* (SpNck2; XP_011668987), and two amino acid sequences coding for SH2- and SH3-domains containing

proteins of *Schistosoma mansoni* (SmaTK5; AAF64151) and *Homo sapiens* (HsFyn; P06241) serving as out-groups. All positions containing gaps and missing data were eliminated. In Planaria, the free-living class of the phylum platyhelminthes, doing BLAST analyses, there was no homolog found. Parasite species names were abbreviated with the first two letters, free-living with the first.

References

1. Felsenstein J. Confidence limits on phylogenies: An approach using the bootstrap. Evolution 1985;39: 783-791.

2. Saitou N, Nei M. The neighbor-joining method: A new method for reconstructing phylogenetic trees. Mol Biol Evol. 1987;4: 406-425.

3. Tamura K, Stecher G, Peterson D, Filipski A, Kumar S. MEGA6: Molecular Evolutionary Genetics Analysis version 6.0. Mol Biol Evol. 2013;30: 2725-2729.

4. Zuckerkandl E, Pauling L. Evolutionary divergence and convergence in proteins. In: Bryson V, Vogel HJ, editors. Evolving Genes and Proteins. Academic Press, New York; 1965. pp. 97-166.
