# Supplementary material for: Evidence for Integrin – Venus Kinase Receptor 1 Alliance in the Ovary of Schistosoma mansoni Females Controlling Cell Survival
Source: PLoS Pathog. 2017 Jan 23;13(1):e1006147. doi: 10.1371/journal.ppat.1006147 (PMC5289644; doi:10.1371/journal.ppat.1006147)
Supplement: S4 Fig — (PDF) [file ppat.1006147.s005.pdf]

Supplementary figure S4: RT-PCR confirming transcripts of SmILK, SmPINCH, and SmNck2 in isolated gonads of *S. mansoni.*

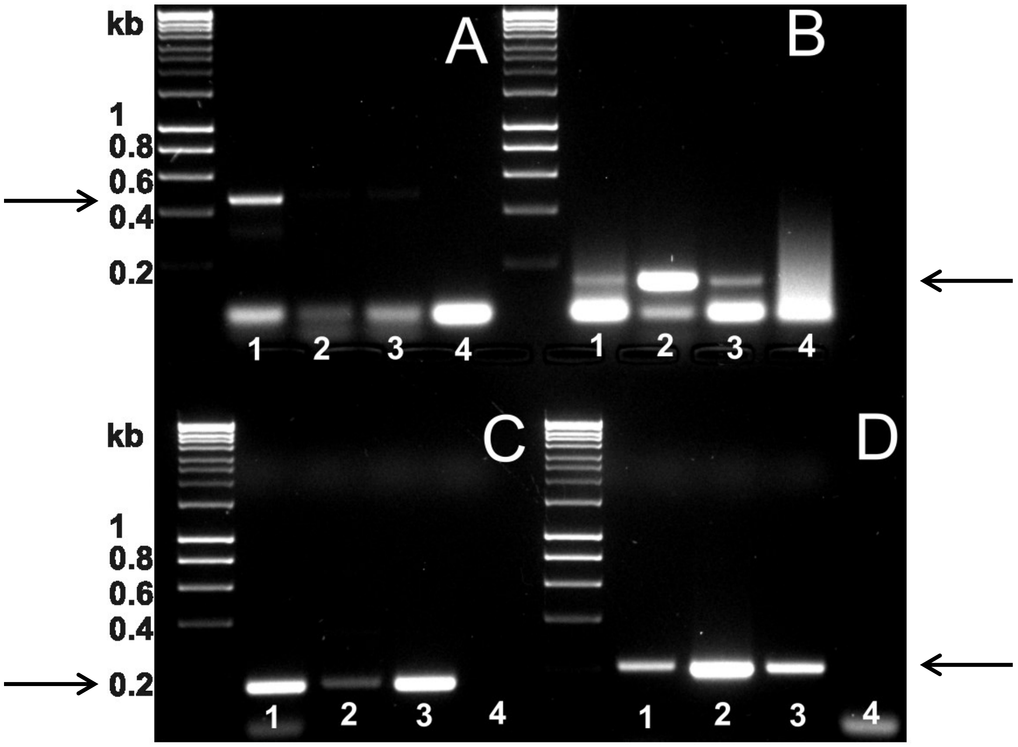

Suppl. Fig. S4

Organ RNA-specific RT-PCRs analyses [1] confirmed the transcription of SmILK (B), SmPINCH (C), and SmNck2 (D) in the ovary (lanes 2) or the testis (lanes 3). Lanes 1 contain cDNA of *S. mansoni* couples as positive controls, and lanes 4 represent RT$^-$ negative controls. In each case amplicons of the expected sizes were obtained (see arrows). For biological control part of NPP-5 was amplified (A), a nucleotide pyrophosphatase/ phosphodiesterase shown before to be predominantly expressed in the tegument and at lower levels in internal tissues [2] but not in the gonads [1]. In this case, amplicons were exclusively obtained with cDNA of couples (lane 1). Parts of SmILK (B), SmPINCH (C) and SmNck2 (D) were amplified and shown to be present in the gonads (lanes 2, 3). In each case (A-D), no amplification products were obtained in the negative controls (lanes 4). Marker, Hyperladder I (Bioline).

References

1. Hahnel S, Lu Z, Wilson RA, Grevelding CG, Quack T. Whole-organ isolation approach as a basis for tissue-specific analyses in *Schistosoma mansoni*. PLoS Negl. Trop. Dis. 2013;7(7): e2336.

2. Rofatto HK, Tararam CA, Borges WC, Wilson RA, Leite LCC, Farias LP. Characterization of phosphodiesterase-5 as a surface protein in the tegument of *Schistosoma mansoni*. Mol Biochem Parasitol. 2009;166: 32-41.
