# Supplementary material for: Evidence for Integrin – Venus Kinase Receptor 1 Alliance in the Ovary of Schistosoma mansoni Females Controlling Cell Survival
Source: PLoS Pathog. 2017 Jan 23;13(1):e1006147. doi: 10.1371/journal.ppat.1006147 (PMC5289644; doi:10.1371/journal.ppat.1006147)
Supplement: S5 Fig — (PDF) [file ppat.1006147.s006.pdf]

Supplementary figure S5: RNAi-mediated knockdown of SmILK.

A

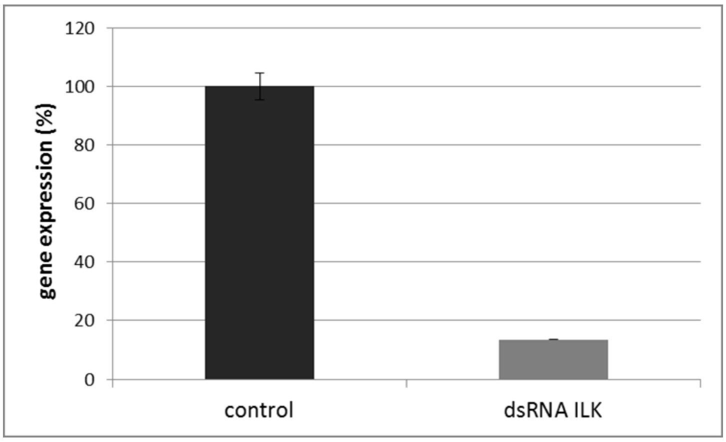

B

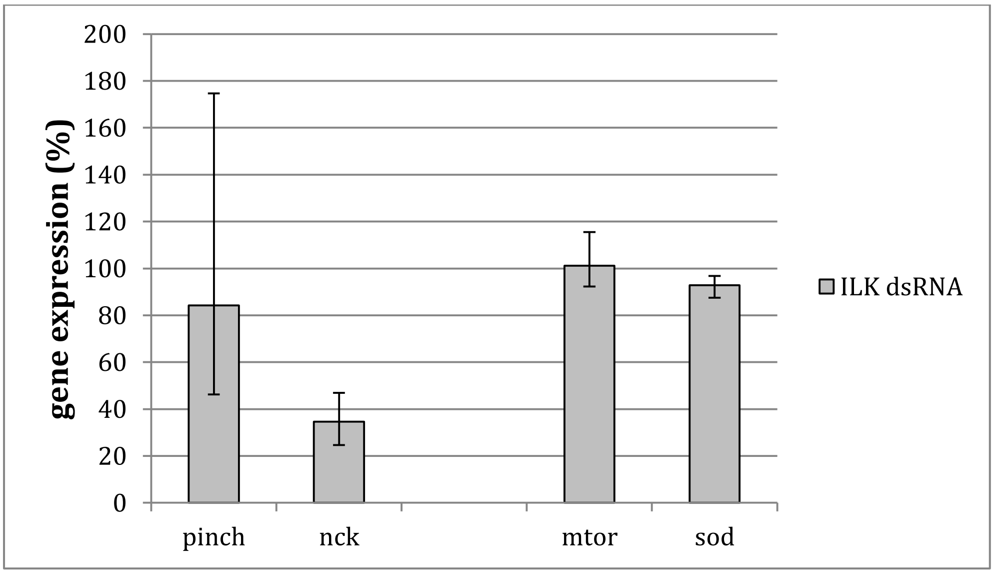

Suppl. Fig. S5

A, RNAi-mediated knockdown of SmILK in adult worms was determined by qPCR analyses (*n = 3*). Using gene-specific dsRNA a reduction of SmILK transcription was achieved down to 13 % of the level found in the control group (no dsRNA), 96 h after electroporation.

B, Results of the qPCR analysis (*n = 4*) to investigate the transcript profiles of biologically related (SmPINCH, SmNck2 = pinch, nck, respectively) and unrelated genes (mtor, Smp_122910, annotated as serine/threonine protein kinase mTOR; sod, Smp_056440, annotated as superoxide dismutase) in females treated with dsRNA against ILK.
