# Supplementary material for: Evidence for Integrin – Venus Kinase Receptor 1 Alliance in the Ovary of Schistosoma mansoni Females Controlling Cell Survival
Source: PLoS Pathog. 2017 Jan 23;13(1):e1006147. doi: 10.1371/journal.ppat.1006147 (PMC5289644; doi:10.1371/journal.ppat.1006147)
Supplement: S6 Fig — (PDF) [file ppat.1006147.s007.pdf]

Supplementary figure S6: Western blot analyses to test the specificity of antibodies

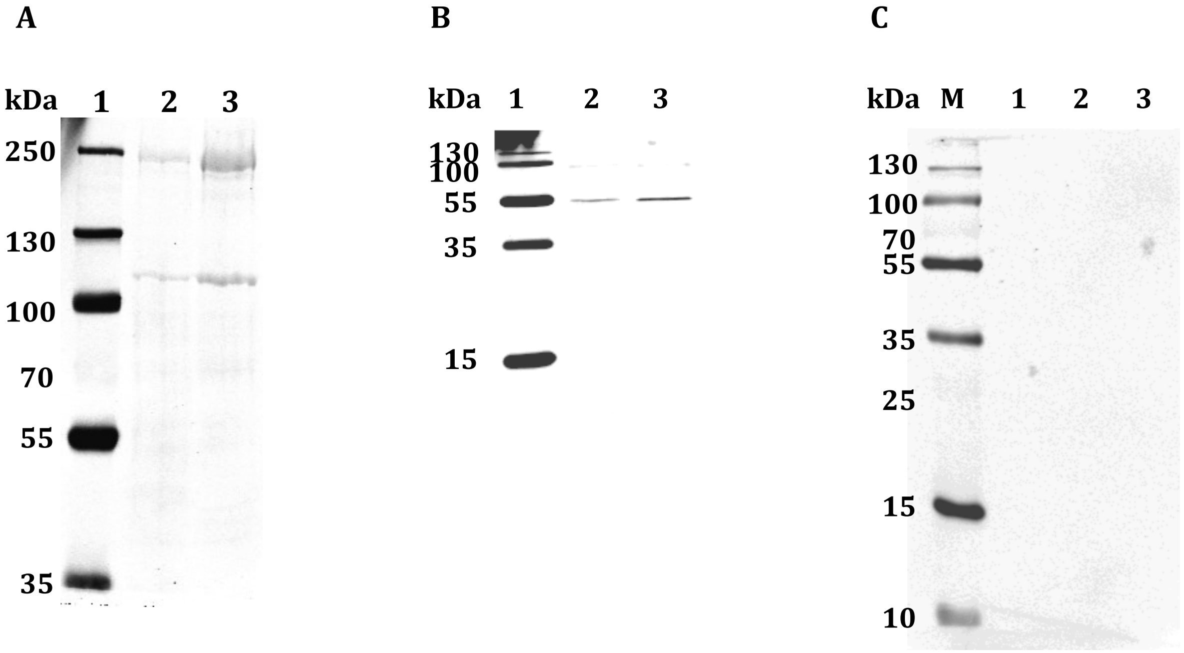

Suppl. Fig. S6

Western blot analyses were performed to test commercial antibodies against laminin (A), tubulin (B), or the secondary antibody (C). Protein lysates of female (lanes 2) or male (lanes 3) adult worms were size-separated by SDS-PAGE (A, 7.5%; B, 15%; C, 12%). All antibodies were used in concentrations of 1 : 5,000. Bands of expected sizes were obtained that represent orthologs of laminin (A) or β-tubulin (B), which according to GeneDB (http://www.genedb.org) are expressed in *S. mansoni* adults and have appropriate sizes (laminins: Smp_148790, Smp_163810; β-tubulins: Smp_078040, Smp_030730). As expected, the secondary antibody showed no cross-reaction against *S. mansoni* protein. Lane 1, protein size marker (Thermo Scientific; PageRuler Plus).
