# Supplementary material for: Evidence for Integrin – Venus Kinase Receptor 1 Alliance in the Ovary of Schistosoma mansoni Females Controlling Cell Survival
Source: PLoS Pathog. 2017 Jan 23;13(1):e1006147. doi: 10.1371/journal.ppat.1006147 (PMC5289644; doi:10.1371/journal.ppat.1006147)
Supplement: S7 Fig — (PDF) [file ppat.1006147.s008.pdf]

Supplementary figure S7: transcript profiles of the pro-apoptotic genes BAK and BAX of *S. mansoni*

A

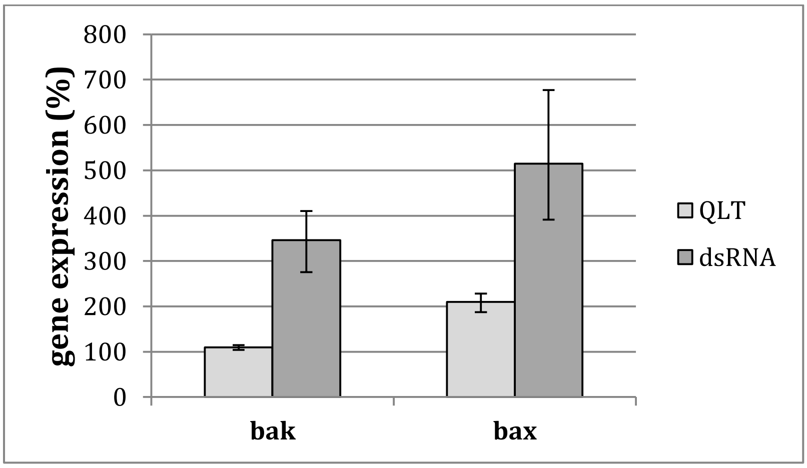

B

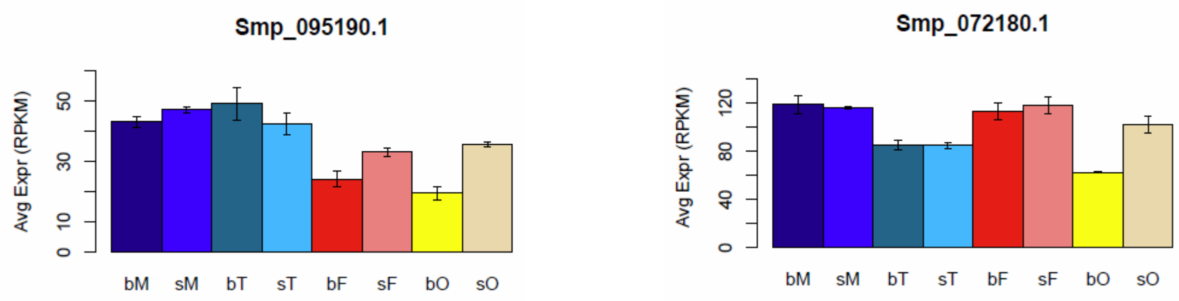

Suppl. Fig. S7

A, qPCR analyses (*n = 4*) of the *S. mansoni* orthologs of the pro-apoptotic genes BAK (Smp_095190) and BAX (Smp_072180) following treatment with QLT-0267 (left columns, light grey) or dsRNA against SmILK (rights columns, grey).

B, result of a comparative RNA-seq analysis [1] in adults and isolated gonads on the basis of normalized, relative expression values (Avg exp (RPKM)) investigating the transcript profiles of BAK (left) and BAX (right) in: bM, bisex (paired) males; sM, single-sex (unpaired) males; bT, testes from bisex males; sT, testes from single-sex males; bF, bisex (paired) females; sF, single-sex (unpaired) females; bO, ovaries from bisex females; sO, ovaries from single-sex females (in each case *n = 3*, except sO: *n = 2*; [1]). One important finding in the context of the paper is that with respect to the expression of both genes sO > bO tendencies were found. This reveals an up-regulation of the pro-apoptotic genes BAK and BAX in ovaries isolated from unpaired, sexually immature females.

References

1. Lu Z, Sessler F, Holroyd N, Hahnel S, Quack T, Berriman M, et al. Schistosome sex matters: a deep view into gonad-specific and pairing-dependent transcriptomes reveals a complex gender interplay. Sci Rep. 201;6: 31150.
